# The Rise of the OM-LoC: Opto-Microfluidic Enabled Lab-on-Chip

**DOI:** 10.3390/mi12121467

**Published:** 2021-11-28

**Authors:** Harry Dawson, Jinane Elias, Pascal Etienne, Sylvie Calas-Etienne

**Affiliations:** Laboratoire Charles Coulomb (L2C), Université de Montpellier, CNRS, 34095 Montpellier, France; pascal.etienne@umontpellier.fr (P.E.); sylvie.etienne@umontpellier.fr (S.C.-E.)

**Keywords:** microfluidic, optofluidic, lab-on-chip (LoC), analysis, detection, manipulation

## Abstract

The integration of optical circuits with microfluidic lab-on-chip (LoC) devices has resulted in a new era of potential in terms of both sample manipulation and detection at the micro-scale. On-chip optical components increase both control and analytical capabilities while reducing reliance on expensive laboratory photonic equipment that has limited microfluidic development. Notably, in-situ LoC devices for bio-chemical applications such as diagnostics and environmental monitoring could provide great value as low-cost, portable and highly sensitive systems. Multiple challenges remain however due to the complexity involved with combining photonics with micro-fabricated systems. Here, we aim to highlight the progress that optical on-chip systems have made in recent years regarding the main LoC applications: (1) sample manipulation and (2) detection. At the same time, we aim to address the constraints that limit industrial scaling of this technology. Through evaluating various fabrication methods, material choices and novel approaches of optic and fluidic integration, we aim to illustrate how optic-enabled LoC approaches are providing new possibilities for both sample analysis and manipulation.

## 1. General Introduction

Since the mid-2000’s, optofluidics has developed into a separate field; independent from its merging parental fields of ‘optics and fluidics’ [1,2]. In summary, these interactions are an exchange of energy and momentum between the first system of light and the second being the fluid. From the less energetic infrared (IR) to the most energetic ultraviolet (UV) light, the manifestation of these interactions can vary widely depending on the energies, and scales of the interacting systems.

In contrast to it’s manifold nature, covering all interactions between optics and fluidics regardless of system scale, optofluidics has two main groups of application that have been identified. As suggested by Monat et al. [2] and later by Hawkins and Schmidt [3], it can be either:

- The case of fluids being driven by light which manifests on the practical level as particles and fluids manipulation.
- Or conversely light manipulation by fluid which mainly manifests as the analysis of biological and chemical samples on the LoC level.

Even though the earliest demonstrations of optofluidics were macro-scale liquid-enabled optical devices [4,5], the field has recently flourished on the grounds of microfluidic approaches. The scale reduction with microfluidic devices provides multiple physical advantages such as economy of reagents, increased reaction rates and a decreased laboratory footprint [6]. These systems enable static, mono and multi-phase flows depending on their application. Multi-phase (droplet) flows being of increasing significance [7], specifically regarding detection, as they act as individual reaction chambers to monitor high-throughput reactions. Optical integration with microfluidics initially enabled novel possibilities such as single molecule manipulation and analysis, with early progress being highlighted in the following reviews [1,8].

Optical “add-ons” have since paved the way for a new generation of LoCs which we will baptise as OptoMicrofluidicLoCs (OMLoCs) for the rest of this review. These optically integrated devices have used photonics to realise essential Lab-on-chip processes such as sample processing, detection and manipulation and through which have made progress to achieving the potential of the predicted LoC paradigm.

However, formation of these OMLoC devices not only combines the fabrication challenges of both microfluidic platforms and optical circuits but requires successful integration within the same device. As such, material compatibility and fabrication complexity are central factors to device development. These issues are usually addressed either with a hybrid approach like combining silicon based photonics and polymer microfluidics [9,10], or with a monolithic approach such as the use of femtosecond laser (FSL) processing of fused silica, and Foturan glasses [11] to create both channels and waveguides. These monolithic approaches are on the rise, as seen with Lithium Niobate [12] and nano-porous materials [13] that demonstrate alternative OMLoC fabrication methods.

In this review, we address the subject of on-chip optical control due to its increasing importance in novel nano-biotechnologies. These approaches have enabled contactless, single and multiple particle manipulation as well as mass transport in order to implement on chip control functionalities. In the first section entitled “On Chip Fluids Manipulation by light” we present on chip implementation of optical forces. Since optical control is usually performed on a chip using evanescent field, we will tackle evanescent field control’s theory, modeling and simulation. Finally, we will break down the work in this field by application type, drawing special attention to the fabrication techniques used, and the order of magnitude of the powers used for manipulation.

Subsequently, we address the second vital LoC process of analysis that is enabled by OMLoC integration. Bio-chemical analytical detection remains essential across multiple sectors, from environmental monitoring to pharmaceutical development. Recent OMLoC integrated approaches have demonstrated increased detection sensitivity [8] while challenging traditional analytical methods requiring external sample preparation, separation and detection [6]. However, miniaturisation of these detection modes requires novel designs to overcome physical constraints that become apparent at the μ-scale. As such, we discuss the main photonic detection methods (absorbance, luminescence and refractive index (RI) detection) and how progress is being made to generate stable and sensitive devices that can be integrated into established workflows.

Through examining recent developments in this field, and taking into account fabrication methods, material choices and fluidic/photonic integration, we aim to assess the progress while highlighting the clear potential of OMLoC’s to realise the aspirations of the integrated LoC.

## 2. OMLoC: On-Chip Fluids Manipulation by Light

### 2.1. Introduction

Fluids and particles manipulation is an essential lab-on-chip function. Biology and biochemistry have both found a great interest in microfluidic based single and multiple particle manipulation, for both fundamental and applied research. Applications may include for example: isolating single particles such as DNA molecules [14] in order to analyse them, being able to manipulate single blood cells’ in order to study their elasticity [15], extracting the least abundant cells for diagnosis of diseases such as cancer [16], and HIV [17].

In a counter-intuitive manner, fluids’ behavior on this scale is marked by the interesting ability to exploit electrokinetic and optoelectronic effects, along with optical and acoustic nano forces, for particle manipulation. They enable the implementation of on chip fluid actuators [18,19], mixers [20,21,22], traps [19,23], and cell sorters [24] for example.

High spatial resolution and adjustable trap size along with low energy requirements and the ease of on chip integration are the most important characteristic that influence the popularity of a manipulation technique. In order to respond to lab-on-chip needs, each one of the above cited techniques has its place in the art. Acoustic manipulation is one of the most nondestructive manipulation techniques. The order of magnitudes of the powers (from $10^{-2}$–$10\;{W/\mathrm{cm}}^{2}$) and the frequencies (1 kHz to 500 MHz) employed are around those for ultrasonic imaging which are safely and widely used in diagnosis [25]. Electrokinetic manipulation remains one of the easiest to implement on a chip through the integration of electrodes [26,27], and optoelectronic tweezers (OETs) are the most configurable, versatile and energy efficient [28]. Conventional optical tweezers (COTs) provide a high degree of spatial resolution but suffer from high energy consumption and the bulkiness of their setup.

Direct optical trapping and actuation are possible using electromagnetic forces acting on metal as well as on dielectric particles. Optical control only became experimentally possible after the invention of lasers in the 60’s. Traditional optical tweezers, or traps are created by using a high numerical aperture (NA) objective to tightly focus a laser beam, in order to create a region in space where a micro-metric particle will experience a force due to transfer of momentum from the scattering of photons. These conventional traps were first demonstrated to be useful for trapping particles, atoms, and molecules by Arthur Ashkin [18,29,30], who recently received the Nobel prize in 2018 for his work on this matter. It was later implemented in the work of many research groups from the early 90’s [31], until the present time [32]. Optical chromatography, appeared a couple of years later and exploited optical forces of loosely focused lasers for cell sorting applications [33,34,35]. Deformation of Liquid-Liquid interfaces was also reported, enabling droplet fabrication, menisci creation [36,37,38] and optically induced cavitation bubbles [39,40,41]. Optical actuation was revealed to be also possible using form birefringence [20].

**Figure 1:**
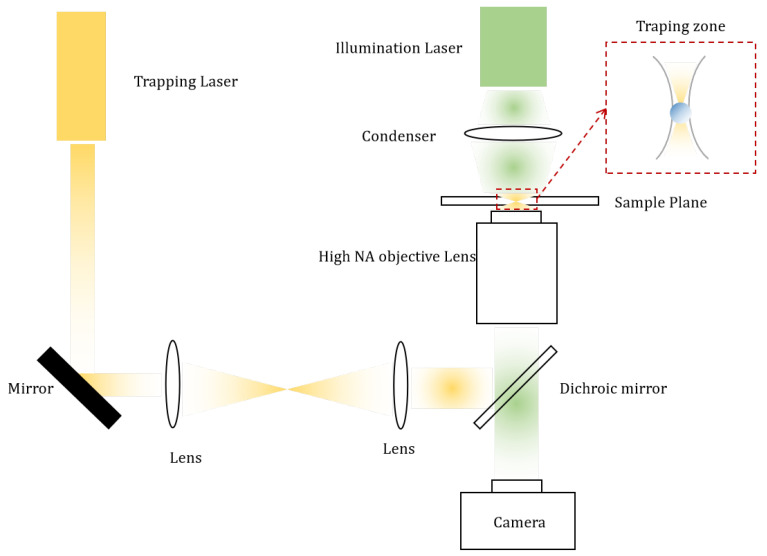
An example of an experimental setup of a traditional optical tweezer.

As seen in Figure 1, the traditional optical tweezers/actuators approach utilizes a bulky and fragile experimental setup, which is expensive and not adapted for full integration on a chip. Also, these devices are only capable of implementing one trap at a time, which limits their throughput. The use of holographic optical tweezers can solve this issue by enabling the implementation of multiple traps at once [42]. Among the disadvantages of far field optical tweezers is the diffraction limit imposed by the NA of the objective and the wavelength resulting in an optically unresolved trap. As a matter of fact, the spatial resolution is usually taken to be 0.61 λ/NA, even for UV light, a lens is limited to the resolutions of around 200 nm [43]. As such, trapping nanometric samples becomes challenging. Needless to say that this setup very often requires high laser power, resulting in undesirable and irreversible thermal damage of biological samples, let alone the high energy consumption of these CTs. For applications dealing with biological samples, IR lasers should be used in order to offer a low damage manipulation of living cells however this will limit the resolution.

**Figure 2:**
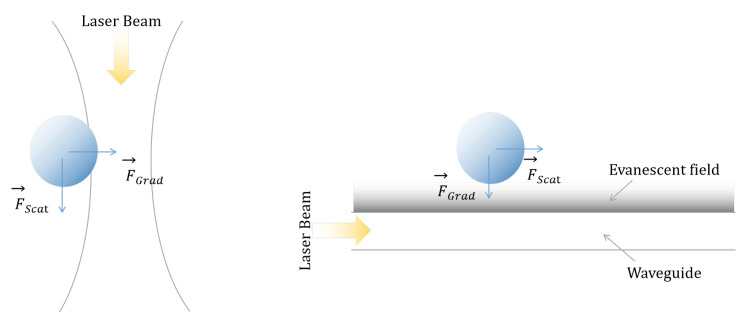
Schematic comparison between far field and near field optical manipulation.

In order to solve the scaling problem as well as the high energy consumption and the full on chip operation, direct optical manipulation has lately found a solution in the evanescent field forces [44], as schematically illustrated in Figure 2. This will be the main focus of the following sections.

Before carrying on the discussion about evanescent field optical forces, it is important to highlight that OETs are also a great alternative to COTs. They indeed do not rely on the same principles as direct optical traps but they have the merit of being briefly addressed. An OET can be seen as an optically induced dielectrophoretic trap. A simple OET setup utilizes a photoconductive material (typically amorphous silicon [45], but also photoconductive polymers such as titanium oxide phthalocyanine [46]), deposited on an indium tin oxide (ITO) coated glass plate, as a lower support, for example. The sample to be manipulated is sandwiched between this lower assembly and another ITO coated glass plate. The lower and upper plates are electrically biased. Through the lower glass plate the photococnductive material is imprinted with an illumination pattern, in order to locally increase the photogenerated carriers thus creating virtual electrodes. An electric field is formed in the liquid above the virtual electrode. The resulting electric field gradient in the liquid layer creates dielectrophoretic force for microparticle manipulation. As such, depending on the difference between the permittivity of the particle and that of the surrounding medium ($\epsilon_{p}-\epsilon_{m}$), this particle perceives either an attractive or a repulsive force, with respect to the virtual electrode. When the illumination is ceased the photosensitive material recovers, and another electrode’s geometry can be imprinted. This makes it one of the most re-configurable manipulation technique, able to create modulable traps as well as size dependent sorters such as the case of the moving comb experiment [19]. Large forces can be achieved with low optical power intensity, enabling the trapping of particles as small as gold nanoparticles, quantum dots and nanowires [47]. Any spatial light modulator can be used for the illumination, as there is no stringent requirements on the beam shape and coherence, unlike the case of optical tweezers. In addition low fabrication cost makes them attractive for disposable systems [28]. OETs utilizing an engineered non-uniform background field were recently reported [48], and were found to be able to enhance the trap resolution without the need to decrease the imprinted electrodes dimensions (i.e., the beam size). Various effects, other than Dielectrophoresis (DEP) trapping occur in OET, they are extensively discussed along with the physical principals of OETs in reference [45].

### 2.2. Theory: Analytical Solutions and Numerical Simulations

Optical control applications may vary, but basically rely on the same governing equations. Simply put, a light beam carrying momentum and energy can be used to directly move, trap, or guide a particle and is subsequently able to deform a liquid-liquid (L2) interface.

**Figure 3:**
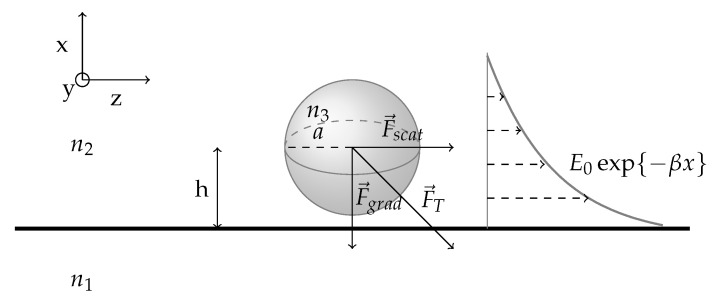
Schematic drawing of a sphere of radius *a* in the evanescent field of an optical waveguide. The evanescent field having a damping factor β and a maximum amplitude $E_{0}$ is expressed as $E_{0}\exp\{-\beta x\}$.

For a better understanding of the mechanisms by which optical forces act on an object, we will present the general equations that can describe a standard but simplified optical manipulation problem on a chip. We consider a spherical particle of radius *a* and refractive index $n_{3}$ in the evanescent field of an electromagnetic wave created by total internal reflection (TIR) on the interface between two medium of refractive indices $n_{1}$ and $n_{2}$ as seen in Figure 3.

For a clear understanding of the underlying physical quantities, analytical equations are necessary along with the decomposition of the force into two components:

(1) a scattering force ${\vec{F}}_{scat}$, in the direction of light propagation
(2) a gradient force ${\vec{F}}_{grad}$, in the direction of the spatial light gradient.

This gives a more direct relation between the force exerted and the experimental parameters such as the laser power and wavelength, the particles dimensions and refractive index as well as the medium’s refractive index.

Let us first of all define the dimensionless size parameter $\alpha =\frac{2\pi a}{\lambda_{2}}$, which is a measure of the size of the particle (represented by it radius *a*) with respect to the wavelength $\lambda_{2}$ of the incoming electromagnetic radiation in medium 2. Three regimes of interaction can be identified based on this ratio of particle dimension to the wavelength of manipulation:

- Rayleigh Regime: for α<<1, hence for particles smaller than the wavelength. The theory for the evaluation of each one of them separately is based on electromagnetic model or what is also called the dipole approximation [49],
- Mie Regime: for α>>1, hence particles larger than the wavelength the calculation can be simplified by the use of ray optics (Some researchers suggest that the Mie formulation can still be applied to values of α around 1 [3]) [50,51]
- Lorenz-Mie Regime: and finally for α∼1, when the particle’s size is comparable to that of the wavelength of light, the generalised and more complex Lorenz-Mie theory, from which the first two regimes decline, must be applied.

#### 2.2.1. Rayleigh Regime

For a spherical particle whose size is much smaller than the wavelength of light, the electric field induces an electric dipole moment in the object that is pulled toward the focus by intensity gradients of the electric field of light. Ng and coworkers have studied Rayleigh particle in the evanescent field of an optical waveguide [52,53].

The time-averaged gradient force, arises from the interaction of the induced dipole with the inhomogeneous field:

(1) $${\vec{F}}_{grad}=\frac{2\pi \zeta }{cn_{2}^{2}}\vec{\nabla }I_{0}$$

(2) $$\zeta =n_{2}^{2}a^{3}\left(\frac{{\left(\frac{n_{3}}{n_{2}}\right)}^{2}-1}{{\left(\frac{n_{3}}{n_{2}}\right)}^{2}+2}\right)$$

where $I_{0}$ is the intensity of the incident light, c is the speed of light in vacuum, λ is the wavelength of the trapping laser and ζ is the polarizability of the sphere. This gradient force is proportional to the intensity gradient, and points either up the gradient when $\frac{n_{3}}{n_{2}}>1$ or down when $\frac{n_{3}}{n_{2}}<1$.

On the other hand these propulsive forces take the form of:

(3) $${\vec{F}}_{scat}=\frac{I_{0}\eta n_{2}}{c}\vec{z}$$

with

(4) $$\eta =\frac{128\pi^{5}a^{6}}{3\lambda^{4}}{\left(\frac{{\left(\frac{n_{3}}{n_{2}}\right)}^{2}-1}{{\left(\frac{n_{3}}{n_{2}}\right)}^{2}+2}\right)}^{2}$$

η is the scattering cross section of the sphere. The scattering force is in the direction of propagation of the incident light and is proportional to the intensity.

As we can see from the above equations that the scattering and the gradient forces respectively scale with $a^{6}$ and $a^{3}$, for particle radius and with $I_{0}$ and $\vec{\nabla }I_{0}$, for input power. Clearly, when the particle radius gets smaller more power and power gradient should be deployed.

#### 2.2.2. Mie Regime

When the Mie scattering conditions are satisfied (α>100 according to Roosen [54], α>80 according to Almaas [55]), the problem can be solved by geometrical optics. Larger objects act as lenses refracting the rays of light and modifying the momentum of photons. Walz [56] has investigated the ray optics calculation of the radiation forces exerted on a dielectric sphere at a distance h of an evanescent field. Keeping in mind that rays in the evanescent field have imaginary refractive angles.

**Figure 4:**
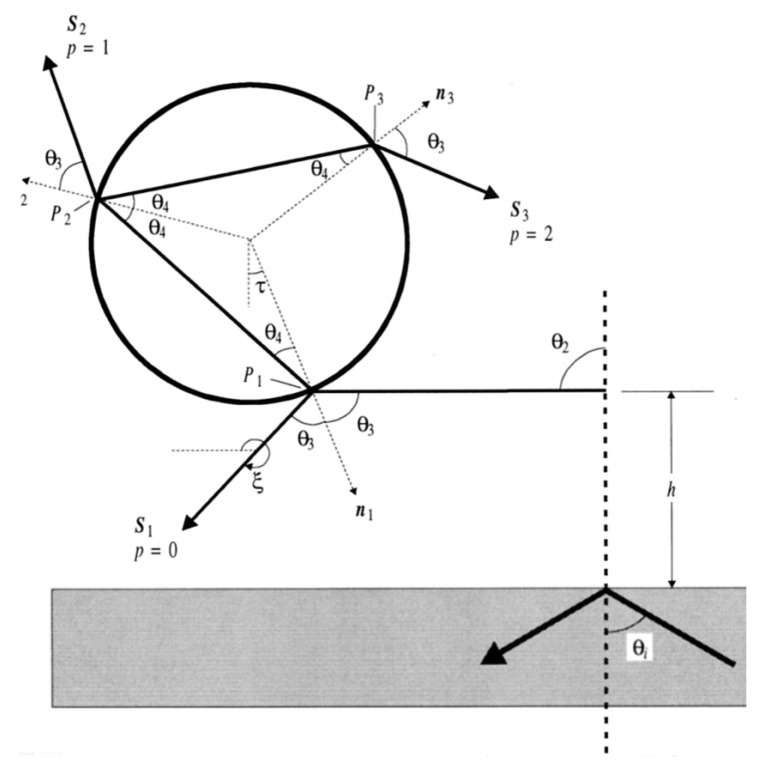
Schematic definition of the variables used in tracing the path of a ray through the dielectric sphere. Reprinted with permission from [56] © The Optical Society.

When rays strike onto the sphere, a fraction of the beam is reflected and the other is coupled into the sphere where it is subject to multiple internal reflections in the sphere itself accompanied by a transmitted fraction at each encounter with the interface as seen in Figure 4.

Complex angles and contact point must be calculated using Snell’s law. After determining these quantities, and as a direct result of the momentum conservation, the total force $\vec{F}$ is determined by the difference between the rate of momentum of the immersing and emerging rays:

(5) $$\vec{F}=\vec{M_{in}^{\prime }}-\vec{M_{out}^{\prime }}$$

The rate of momentum contained in a propagating ray of light is related to the power of the ray P as:

(6) $$\vec{M^{\prime }}=\frac{Pn_{2}}{c}\vec{k}$$

$\vec{k}$ is the unit vector pointing in the direction of propagation of the ray.

As a summary we can see that in the Mie regime, the force exerted on a particle depends only on the power contained in the beam and that the refractive index of the medium has a negligible effect on the motion of the particle being studied.

#### 2.2.3. Lorentz-Mie Regime

For a particle whose size is comparable to the wavelength, no analytical expression of the force can be deduced and the general electromagnetic formalism should be applied in a simulation context.

Multiple software/programming language were used in this context: Almaas and Brevik used Fortran for their simulation [55] while Hellesø et al. used RF-toolbox of Comsol [57] and Matlab [58] as well.

**Figure 5:**
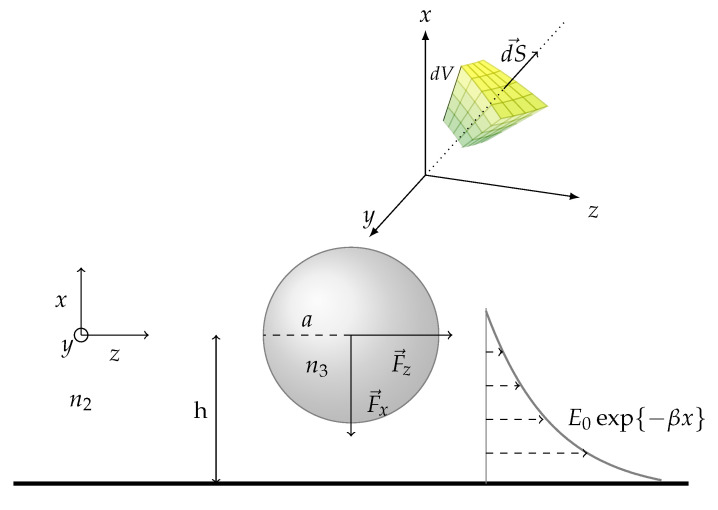
Schematic drawing of a sphere of radius *a* in the evanescent field of an optical waveguide.

As ingeniously explained by Novotny [44] the optical force $\vec{F}$ acting on the surface S of an object can be computed by integrating either the volume force density $\vec{f}$ over the total volume or by integrating the Maxwell stress tensor (MST) over the surface of the sphere:

(7) $$\vec{F}=\oint_{V}\vec{f}dV=\oint_{S}T_{ij}\vec{dS}$$

where $\vec{f}$ is the volume force density, and $T_{ij}$ is the MST. dV is the elementary volume of the particle and $\vec{dS}$ is the unit vector normal to the elementary surface dS and pointing outward with respect to the sphere as represented in Figure 5.

$\vec{f}$ in a charge-free nonmagnetic medium, without electrostriction, can be found as the divergence of Maxwell’s stress tensor and can be expressed as:

(8) $$\vec{f}=-\frac{1}{2}E^{2}\vec{\nabla }\epsilon_{2}$$

where $\epsilon_{2}$ is the permittivity of the surrounding medium and E the electric field decaying exponentially with the penetration depth along the *y* axis:

(9) $$E=E_{0}\exp\{-\frac{2\pi }{\lambda_{1}}\sqrt{(\sin{\theta_{1}}^{2}-{\frac{n_{2}}{n_{1}}}^{2}})x\}=E_{0}\exp\left\{-\beta x\right\}$$

With $E_{0}$ being the amplitude of the electric field, $\lambda_{1}$ and $\theta_{1}$ being respectively the wavelength and the angle of incidence in the medium 1 and finally β being the damping factor of the evanescent wave. The MST $\left[T_{ij}\right]$ represents the density of momentum of the field. It can be written according to the definition proposed by Minkowski in the form:

(10) $$\left[T_{ij}\right]=\left[\epsilon_{2}{\vec{E}}_{i}{\vec{E}}_{j}^{*}+\mu_{2}{\vec{H}}_{i}{\vec{H}}_{j}^{*}-\frac{1}{2}\delta_{ij}\left(\epsilon_{2}{\vec{E}}_{k}{\vec{E}}_{k}^{*}+\mu_{2}{\vec{H}}_{k}{\vec{H}}_{k}^{*}\right)\right]$$

$\epsilon_{2}$ and $\mu_{2}$ denotes the permitivity and the permeability of the surrounding environment (medium 2), and H the magnetic field. The notation ${}^{*}$ designates the conjugate complex.

Almaas and Brevik [55] have applied this above explained method to the case of a micrometer sized spherical particle in the evanescent field of a waveguide. Their simulation considered a laser power of 150 mW and multiple combinations of refractive indices $n_{2}$, $n_{3}$ (while keeping $n_{1}$ at 1.75) for both the parallel (p) and perpendicular (s) polarisation. Their results were presented as plots of nondimensional forms of $F_{z}$ and $F_{x}$ as a function of the size parameter α. They all converged to the following explanation for all the combinations used: $F_{x}$ being always negative meant an attractive force between the substrate and the sphere and $F_{z}$ being always positive meant that the sphere was being propagated along the positive z direction. As a rule of thumb the interaction between the sphere and the field is the strongest when the wavelength is of the same order of magnitude as the sphere diameter.

**Figure 6:**
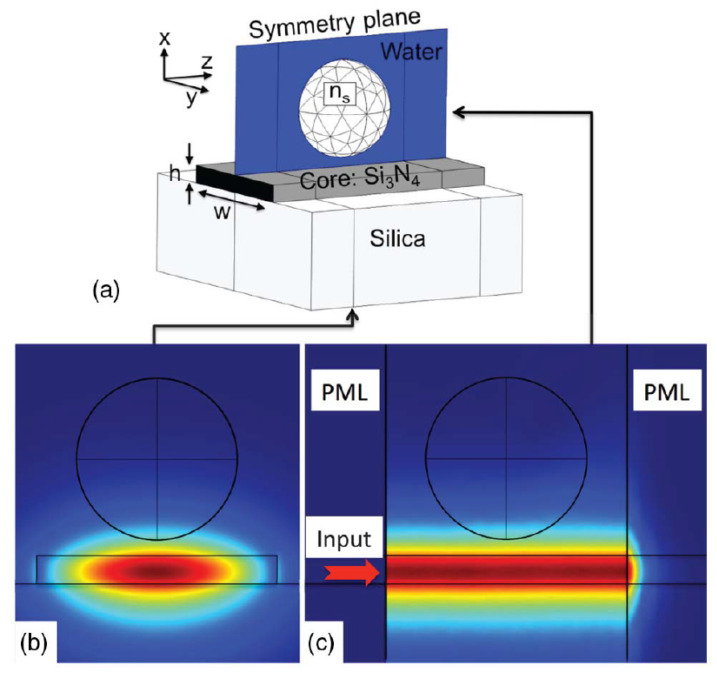
Outline of the model by Hellesø with (**a**) a sphere 100 nm above strip waveguide and (**b**) the fundamental TE mode used to excite the waveguide with 1 mW power as shown in (**c**) as well. Reprinted with permission from [59] © The Optical Society.

A more recent and elaborate work done by Hellesø [59] considered a dielectric sphere in the evanescent field of a waveguide as illustrated in Figure 6.

Instead of integrating the Maxwell stress tensor on the surface of the sphere they expressed *f* as a function of a quantity called the Abraham-Minkowski surface pressure σ:

(11) $$\sigma =-\frac{1}{4}\epsilon_{0}\int_{-a}^{a}\left(\right|E_{t}{|}^{2}+|E_{r}{|}^{2})\frac{d\epsilon }{dr}dr$$

With $E_{t}$ and $E_{r}$ being the amplitudes of the tangential and radial components of $\vec{E}$ with respect to the surface of the sphere. By integrating the local pressure on a particle, the optical force on the particle can be found, giving an alternative to using the MST.

**Figure 7:**
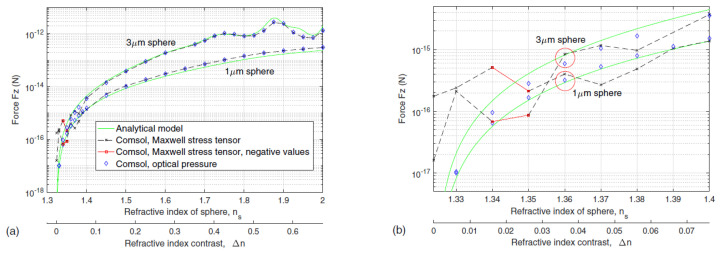
A comparison between MST and optical pressure for simulation: the computation of the radiation force $F_{z}$ on 1 and 3 μm spheres for (**a**) $n_{water}<n_{s}<2$ and (**b**) $n_{s}<1.4$, that is, zoom of (**a**). Reprinted with permission from [59] © The Optical Society.

Even though they are mathematically equivalent, the simulation results showed a better agreement with the Almaas and Brevik model (which they named the analytical model) especially for smaller index contrast between the medium and the sphere as seen in the comparison in Figure 7. Their model introduced a 100 nm gap between the particle and the interface. The wavelength used for the simulation was 1070 nm, with a very low input power of 1 mW.

Neither of all the above cited works have considered back coupling between the sphere and the surface. Nonetheless, the study in reference [60] found an interest in considering this phenomenon. Their findings concerning the scattering force were similar to the other studies. But they found a gradient force switching from attractive to repulsive as the particle approaches the interface which explains the vertical particles’ oscillation in Kawata’s early works [31]. They also observed that this coupling is not important for a distance *d* larger 0.2a.

It was also theoretically and experimentally confirmed that a phenomenon observed in nanophotonic cavity and called self-induced back-action (SIBA), can be used to enhance the trapping without the need to locally enhance the electromagnetic field [61,62]. In SIBA trapping, the particle’s motion couples to the resonance frequency of the cavity, resulting in a build up of intra-cavity intensity, and thus the enhancement of the optical force exerted.

#### 2.2.4. The Trapping Potential and Particle’s Dynamics

In the case of a particle trapping problem and after computing the trapping force, accurate estimation of the trapping potential is of significant importance. Mainly because the trap stiffness, the trapping range (hence the traps spacing), particle’s location and other characterizing parameters of an optical trap are extracted from the trapping potential. A near-field optical trap generates a force-field that can be decomposed into a conservative/irrotational component and a non-conservative/solenoidal component, using the Helmholtz-Hodge decomposition (HHD) [63]. HHD can be applied in the case of a sufficiently smooth force $\vec{F}$, defined in a bounded domain Ω with a smooth boundary δΩ and results in the following decomposition:

(12) $$\vec{F}=\nabla \Phi +\nabla \times \vec{A}$$

where Φ is the scalar trapping potential and $\vec{A}$ is the vector trapping potential. For a conservative field, $\vec{\nabla }\times \vec{F}=\vec{0}$, the trapping potential is the scalar potential Φ that can be calculated using a line integral of the field vector. When the trapping force is not purely conservative (i.e., has a solenoidal component, i.e., $\vec{\nabla }\times \vec{A}\neq \vec{0}$ ), this conventional method gives faulty results. In such case the potential Φ can be calculated by solving the following equations:

(13) $$-\nabla^{2}\Phi =\nabla \vec{F}\;\mathrm{on}\;\Omega$$

(14) $$\nabla \Phi \cdot \vec{n}=\vec{F}\cdot \vec{n}\;\mathrm{on}\;\delta \Omega$$

Equation (14) is a partial differential equation with Neumann boundary conditions that can be numerically solved. It was confirmed that in the case of plasmonic traps such as a C-shaped, or Archimedean spiral engraving in a gold film and cylindrical gold nanopilar, the force-field has non-negligible solenoidal component and thus cannot be considered as a purely conservative field [63,64,65]. Therefore, the use of HHD becomes necessary to derive the trapping potential Φ with accuracy.

For particle having small mass *m* the thermal velocity $v_{th}=\sqrt{\frac{k_{B}T}{m}}$ may be observable [66]. This would imply that there is a need to include a stochastic force, that is due to thermal fluctuation, in addition to the other forces acting on the particle. In the case of nanoparticles suspended in a liquid medium and subject to optical forces from a nearby trap, this Brownian motion due to thermal agitation/ random collisions with the fluids molecules should be taken into account, in addition to the drift motion due to the trapping force.

In order to know the position probability density function (PDF) P(r,t) of a particle subject to a drift forces and other random forces such as a thermal agitation, multiple methods have been investigated:

- the approximation of PDF from Brownian dynamics simulation of a sufficiently large number of independent trajectories,
- the resolution of the Flokker-Planck first order differential equation,
- the approximation of P(r) at equilibrium ($t\overset{}{\to }\infty$), by the use of a Boltzmann distribution knowing the field potential.

The first two method are always applicable and they enable us to compute *P* as well as its evolution from t=0 until equilibrium. The third method is usually known to be inaccurate in the case of a non conservative force field. We are going to address each of these methods separately in the rest of this paragraph.

The most common approach of analyzing the nanoparticle motion is to numerically solve the Langevin equation, in order to analyze its Brownian trajectories [67,68,69]. This would imply that in order to find the average behavior of particles, the simulation should be repeated for a large number of particles. Therefore, this approach is computationally intensive. For low Reynolds number, a modified Langevin equation can be derived [69]:

(15) $$\vec{r^{\prime }}\left(t\right)=\frac{D(\vec{r})}{k_{B}T}\vec{F}\left(\vec{r},t\right)+\sqrt{2}D_{\frac{1}{2}}\left(\vec{r}\right)\vec{W}\left(t\right)$$

*r* is the position at the center of the particle, $k_{B}$ is the Boltzmann constant, *T* is the temperature, D is the diffusion tensor. $D_{\frac{1}{2}}$ is obtained by taking the square root of each element of D. $\vec{W}\left(t\right)$ is a vector white noise term. Each cartesian component of $\vec{W}\left(t\right)$ is a Gaussian distribution with zero mean and unit variance. The diffusion tensor includes terms quantifying the interaction between the nanoparticle and the underneath trap surface.

In order to avoid calculating all the trajectories for a large number of molecules, other methods exist, such as the equation first derived by Fokker and Planck. That is a differential equation for the distribution function, describing a system subject to a drag force such as a trapping force and to diffusion ($D=k_{B}T/\gamma$ with γ being the friction coefficient). More precisely this equation can model the position PDF of a particles after a certain time *t*, given as an input a random initial PDF. We are not going to express the Fokker-Planck equation here, details can be found in references [66,70]. Among other cases, the Fokker-Planck equation is applicable to the case of a Brownian particle in the vicinity of an optical trap [70].

The third method is the use of a Boltzmann distribution, to model the trapping of particles, in the case of a conservative force-field. The PDF $P(\vec{r})$ depends on the potential function:

(16) $$P\left(\vec{r}\right)=A_{N}\exp\{-\frac{\Phi (\vec{r})}{k_{B}T}\}$$

where $A_{N}$ is a normalisation factor.

It was confirmed that if the HHD derived field force is considered, this Boltzmann distribution that is usually faulty in the case of non conservative force, gives a very close particle distribution as the Brownian model [63,64]. Despite the presence of a solenoidal component, only the conservative component determines the PDF. Thus it seems logical that the distribution follows the statistics of a purely conservative field.

### 2.3. 2D Optical Manipulation: Actuators

Near field forces were first demonstrated in the early 90’s as an alternative to far field optical control. They have since been used for actuation and sorting of particles. A set of experiments by kawata and coworkers [31] showed that an evanescent wave created under TIR over the surface of a high refractive index prism can manipulate micrometer sized particles with the power of 150 mW at 1.06 μm. Later they demonstrated this using an optical waveguide, thus laterally trapping Polystyrene (PS) spheres with diameters of 1–27 μm, and then longitudinally driving them along the direction of the waveguide channel using an effective power of 80 mW at 1045 nm [71].

**Figure 8:**
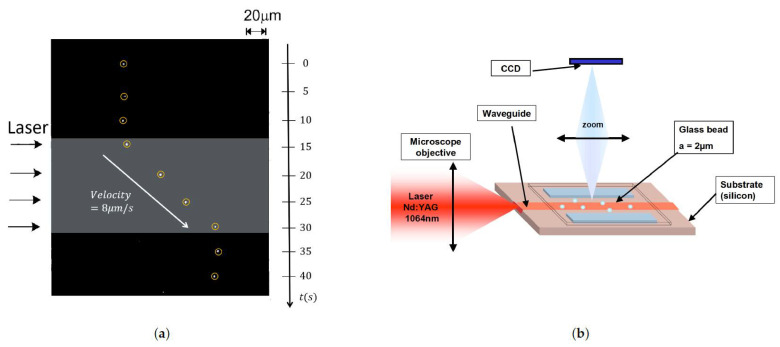
Examples of near field actuators: (**a**) a time lapse showing the propulsion of gold nanoparticles (highlighted in yellow circles), at a velocity of 8 μm/s. Adapted, with permission from [53] © 2002 Elsevier, (**b**) the setup for the manipulation of dielectric nanoparticles on the surface of a silicon nitride waveguide. Reprinted with permission from [59] © The Optical Society.

In the 2000s, a series of papers by Ng et al. [52,53] demonstrated for the first time the propulsion of nano sized particles on the surface of a channel waveguide as seen in Figure 8a. Gold nanoparticles of diameter ranging from 10 nm to 23 nm were used (some researchers may place this case in the realm of what they called direct plasmonic manipulation [72]). In an effort to further decrease the power used for such manipulation, only a 20 mW power IR laser beam was used for the manipulation of cells and dielectric particles on the surface of silicon nitride waveguides [73]. This simple setup is schematically illustrated in Figure 8b.

**Figure 9:**
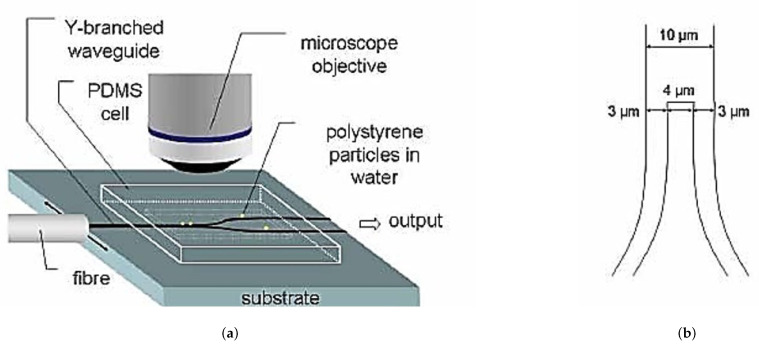
(**a**) the setup of a Y branched sorter and (**b**) the waveguide structure and widths of each branch. Reprinted with permission from [74] © The Optical Society.

Also, PS microparticles were guided by the evanescent field of a more strongly illuminated, output of a Y branched waveguide produced by Cs+ ion-exchange in glass [74]. An input laser power of 165 mW was used in this experiment as illustrated in Figure 9.

**Figure 10:**
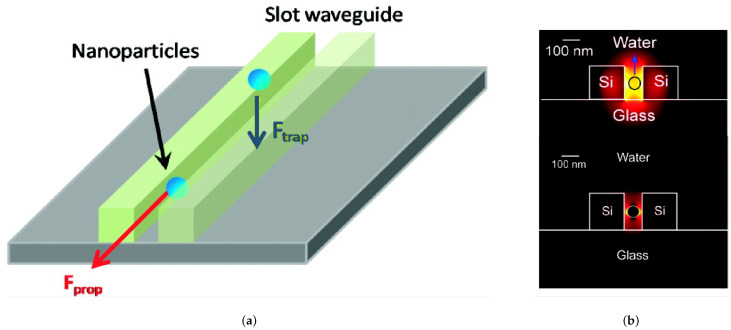
(**a**) Schematic illustrating the transport in a slot waveguide and (**b**) the mode profile for a SOI slot waveguide. Reprinted with permission from [77]. Copyright © 2009 American Chemical Society.

In order to compensate for the high energy requirements, especially when manipulating nanoparticles, slot waveguides were found to be good candidates because they exhibit a strong field confinement [75] as illustrated in Figure 10. The structure consists in a nanometer sized low refractive index slot between two region with high refractive index. The whole structure is surrounded by a cladding with low refractive index. A standard single mode silicon waveguide with a sub-wavelength slot (usually between 50 nm and 120 nm wide) cut through the middle can concentrate the optical energy in the liquid core region of the waveguide [76]. This setup was used to manipulate 75 nm diameter dielectric particles as well as DNA molecules with a input power of less than 300 mW. Additionally, it could extend to even smaller particle sizes of around 10 to 20 nm as per their calculation [77].

For a low cost yet high-performing device, an actuation setup formed with an SU-8 epoxy-based photonic structures, combined with poly(dimethylsiloxane) (PDMS) microfluidics on a fused silica substrate was used by the pioneers Schmidt and Erickson [78]. This system however suffers from high guiding losses, preventing it from being used for complex devices fabrication such as those fabricated with silicon based platforms.

### 2.4. 3D Optical Manipulation: Traps and Tweezers

**Figure 11:**
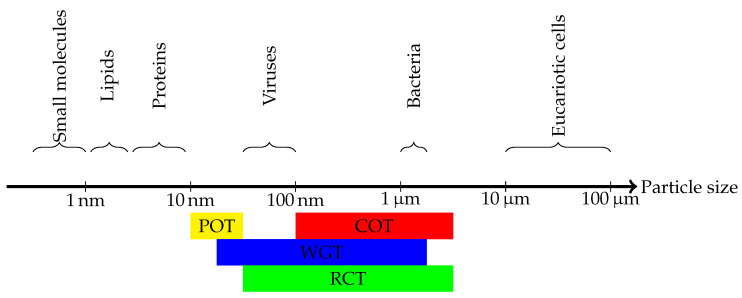
A scale roughly showing micro and nanometric organisms with the most convenient optical trapping techniques.

Optical traps are of a great interest to LoC applications as they enable contact-less and non-invasive single particle manipulation along a wide range of particles size (Figure 11). Due to the multiple inconveniences of traditional optical tweezers [79], the experiments of Kawata and coworkers [31,71] paved the way for near field optical tweezers. Apart from COTs more sophisticated trapping platforms were later introduced, among which we cite: optical fiber and waveguide traps (WGTs) [80], resonant cavity traps (RCTs) [81] and surface plasmonic optical tweezers (POTs) [82,83].

#### 2.4.1. Waveguide Traps (WGTs)

**Figure 12:**
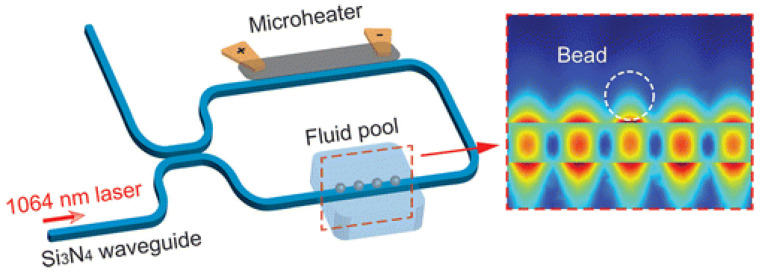
To the left is a schematic of the core components of a nSWAT device. To the right is a cross section of the electric field profile from a 3D full-wave electromagnetic simulation in the presence of a 349 nm polystyrene bead trapped at one of the antinodes of the standing-wave. Reprinted with permission from [85]. Copyright © 2016 American Chemical Society.

Waveguide 3D trapping was successfully implemented using standing waves [84]. This device called nanophotonic Standing Wave Array Trap (nSWAT), was fabricated using silicon waveguides on a silicon-on-insulator (SOI) platform, with a microfluidic layer formed by exposing the Si waveguide in the buried in the oxide. nSWATs were also realised using silicon nitride waveguides as illustrated in Figure 12. After the laser passes through a 50:50 splitter, two counter-propagating waves form a standing-wave in the fluidic pool region, creating hotspots at the antinodes of the standing wave, to which the bead are attracted.

Recent work also demonstrated the versatility of near field mode beating in trapping, actuating and positioning nanoparticles along a few mode waveguide [32]. Depending on the position of the fiber with respect to the waveguide tapered entry, on can excite different modes an subsequently create different functionality.

More recently, with the emerging use of femtosecond laser processing, monolithic chips for manipulation were fabricated in glass [86]. A continuous wave ytterbium fiber laser, at 1070 nm, is used as a light source. Trapping is achieved with an estimated optical power at each waveguide output of about 20 mW. This setup however consisted of two counter propagation beams and even though is not an evanescent field trap, it is worth mentioning.

#### 2.4.2. Resonant Cavity Traps (RCTs)

**Figure 13:**
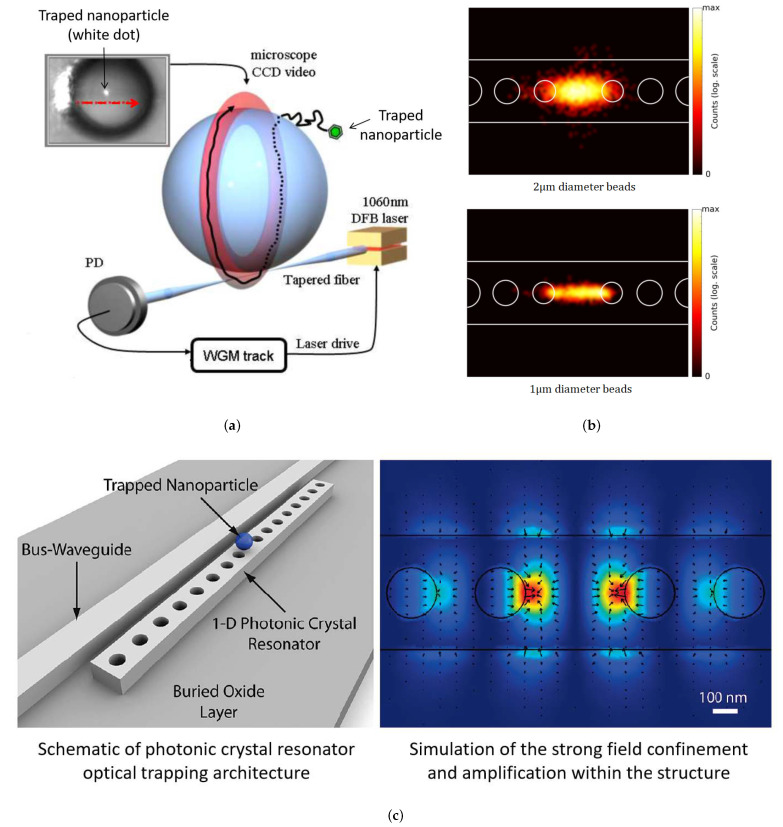
Examples of resonant cavity traps: (**a**) Nanoparticle navigating in the direction that light takes within the WGM. The red arrow represents the light path in the cavity. Adapted with permission from [87] © The Optical Society. (**b**) A count of particles position in a photonic crystal resonator for different bead diameter. Adapted with permission from [89]. Copyright (2015) American Chemical Society. and (**c**) photonic crystal resonators architecture and field confinement. Adapted with permission from [81]. Copyright (2010) American Chemical Society.

The strength of optical traps can be improved by exploiting the field amplification within an optical resonator. For example, the trapping of 48 nm and 62 nm dielectric nanoparticles is demonstrated by exploiting the stationary wave within a photonic crystal resonator [81] (Figure 13c). Arnold et al. [87] demonstrated the trapping and circumnavigation of PS nanoparticles as small as 280 nm in diameter in a circular orbit around whispering gallery mode (WGM) resonators, with a power of around 10 to 50 μW (Figure 13a). Silicon photonic crystal nanocavities can be used to trap 500 nm to 2 μm beads as well as bacteria between the two main anti-nodes located in the central part of the cavity (Figure 13b) with a laser power of 31 mW [88,89].

#### 2.4.3. Plasmonic Optical Traps (POT)

**Figure 14:**
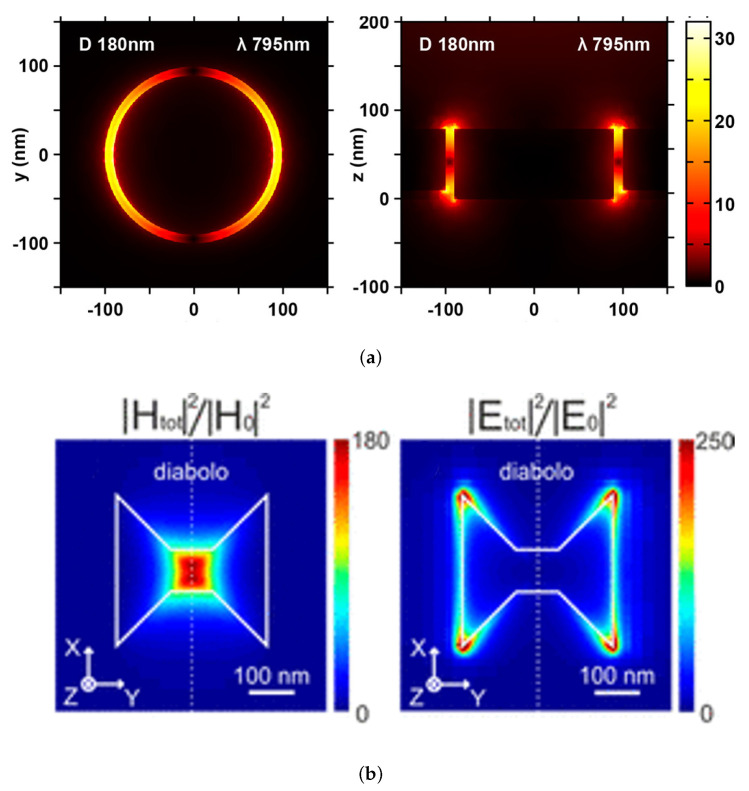
Field confinement in plasmonic nano-traps (from left to right): (**a**) Horizontal (taken 1 nm below the metallic surface) and vertical electric field distributions for a 180 nm inner diameter aperture. Reprinted with permission from [90]. Copyright (2018) American Chemical Society. (**b**) Normalized magnetic and electric intensity distributions in the case of the diabolo nano-antenna. The distributions are plotted in an xy-transversal plane, 10 nm away from the antennas. Adapted with permission from [91]. Copyright (2015) American Chemical Society.

Plasmonic traps use the unique optical properties of metallic nanostructures to enable the guidance and manipulation of light at the nanoscale using the collective oscillations of delocalized electrons of a metal, called plasmons. Surface plasmons are waves that propagate along the surface of such metal. As seen in Figure 14, these plasmonic nanostructures can confine light into subwavelength volumes. which offers a promising alternative to traditional optical tweezers to overcome the diffraction limit and the high energy consumption.

The first plasmonic optical trap was proposed by Novotny with the intensity gradient generated by the strong evanescent field around a 5 nm radius metal tip [92]. Various nanostructures have since been used for optical trapping, such as isolated nanoparticles [93], nanogap antennas [94,95] and nanostructured films [96] to trap particles using typically a couple of hundreds of mW, extremely lower powers than COTs.

Grigorenko et al. [94] utilized the strongly enhanced and localized optical near-fields of closely spaced metallic nanostructures. Optical trapping of nanoparticles and proteins with resonant coaxial nanoaperture using 10 nm Gap was demonstrated with laser power of only 4.7 mW [90]. PS nanoparticles in water were trapped using Au nano-antennas with a laser power of only 5.5 mW [97]. SIBA optical trapping was capable of stabilizing 50 nm nanoparticles, within a subwavelength gold nanoaperature, with less than 2 mW of total power [61].

A concise review article on plasmonic optical trapping and actuation [98] can offer a good first insight into this particular case. In another review on nanoscale optical manipulation [72] a special attention was accorded to the plasmonic effects exploited for particle trapping.

### 2.5. Deformation of Liquid-Liquid (L2) Interfaces and Membranes

Throughout the literature, optical trapping and actuation have been the main focus of researchers. Deformation of soft interfaces remains out the spotlight in spite of many practical application. On the fundamental level, optical bending of the meniscus of a phase-separated liquid mixture induced by the radiation pressure was observed and thoroughly studied by Casner and Delville [36,37,38].

**Figure 15:**
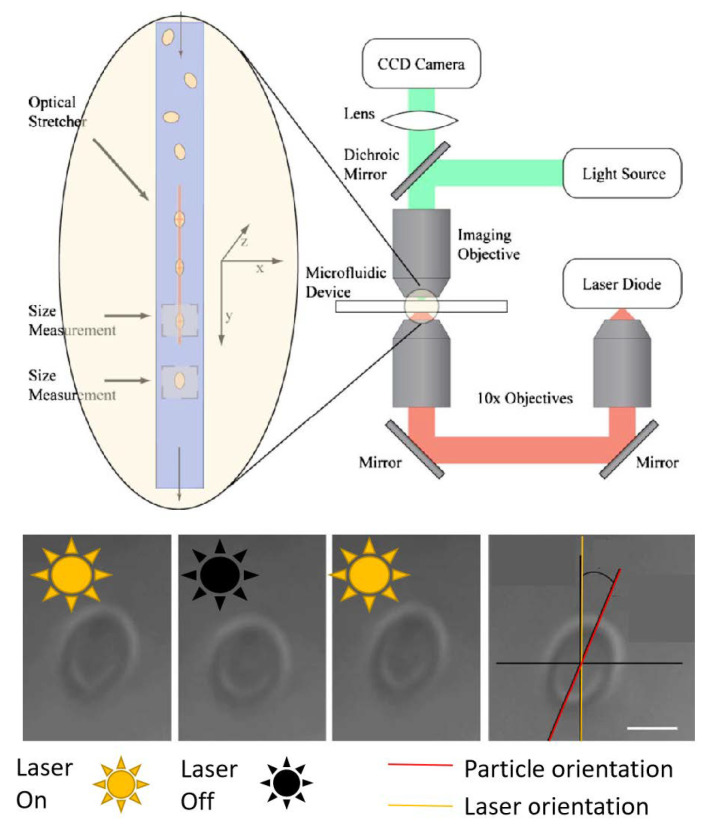
Near field optical forces applied to studies on the elasticity of RBC’s: sequential images of an RBC repeatedly stretched with laser diode. Scale bar = 4 μm. Adapted from [103], SPIE digital library. Open access.

On the practical level, since the primary method for disease (such as cancer) diagnosis remains morphological change in suspect tissue, deformability of cells can be used to locally probe mechanical properties of soft biological systems [99,100]. Optical deformability is found to be a cell’s fingerprint and can be useful as cell marker [101], especially in the case of red blood cell (RBC) [102]. Since cellular mechanical properties of RBC may provide a direct route to detecting diseases, this method was used to study the elasticity of RBC in a contactless manner [15,103] as seen in Figure 15.

Traditional optical tweezers have demonstrated the ability to deform cells using silica beads attached to the membrane as local handles [104,105,106]. Another standard technique is based on the counter propagating beams’ optical trap by Ashkin [18]. Light power as high as 800 mW in each beam can be used, which lead to surface forces up to hundreds of pico-Newton, while avoiding thermal heating. The stretching of the cells occur parallel to the beam propagation.

More recently [107] Martinez et al. used a monolithic optical stretcher fabricated in a commercial microfluidic chip (Translume) by direct implementation of optical waveguides through femtosecond laser writing. These waveguides are perpendicular to the flow of the RBC cells and they host two counter-propagating beams (25 mW each) that will carry out the deformation when the laser power is increased to 1.2 W for 5 s in order to stretch the cell. Other have implemented using the same principles a device to asses the mechanical properties of white blood cells as well [108].

In order to avoid cell damage and to attain a high throughput measurement, several near field approaches were used, nonetheless near field for interface deformation remain less present in the literature when compared to other implementations of optical manipulation. A diode-bar optical stretcher was used [103,109] in order to trap and study the elasticity of RBC. As we can see in Figure 15, the implementation of this system consist of casting the image of a diode bar onto the lower surface of a glass slide on top of which a PDMS microfluidic pool was bonded. Due to the refractive index mismatch between the medium and the interior of the cell, an optical force is induced on the interface. This interaction deforms the cell until its elasticity balances applied optical forces which permits to calculate the elasticity. Ahluwalia’s work on RBCs elasticity offers a better version of on chip manipulation [15,110]. It was demonstrated that the intensity gradient at the edge of narrow waveguides can be used to deform and squeeze cells. Their chip consisted of a tapered waveguide structure made of tantalum pentoxide (${\mathrm{Ta}}_{2}O_{5}$) on oxidised silicon substrate on top of which a thin chamber made of PDMS contains the RBC and is covered with a coverslip.

### 2.6. Summary

Optical manipulation has proved to be irreplaceable by other forms of manipulation due to its versatility, resolution and ability to manipulate nanometric particles. In addition, it can easily be integrated through the mounting of a microfluidic chip on a photonic circuit, in order to exploit near field forces.

From here, the review aims to similarly highlight the developments facilitated by optical OM-LoC pairing, resulting in increasingly sensitive and stable analytical methods. As such, these methods provide the secondary and equally essential function of the LoC approach following sample manipulation.

## 3. OMLoC: On-Chip Optic Enabled Fluidic Analysis

As discussed, on-chip optical integration with microfluidics has multiple analytical advantages, such as increased sensitivity, non-destructive analysis and reduced reliance on expensive laboratory analytical equipment [111]. Resultantly, efforts have been made in recent years to integrate the main photonic analytical methods (absorbance, fluorescence, chemiluminescence and RI detection), among others, within these μ-scale devices [3,8,112].

However, these optical detection methods have varying physical constraints that can limit their integration and resultant analytical performance. For instance, the limit of detection (*LOD*), meaning the minimum analyte concentration that can be positively identified within a sample with a stated confidence level is dramatically altered by optical integration efficiency. The *LOD* is commonly calculated through determining the significant difference between an analyte signal and the standard deviation of the signal blank, as seen in Equation (17).

(17) $$LOD=Y_{B}+3s_{B}$$

LOD determination by comparing the signal to noise ratio; $Y_{B}$ being the mean signal of the blank and $s_{B}$ being the standard deviation of the signal blank [113].

Generally the blank standard deviation ($s_{B}$) is accepted at a factor of 3, however can rise to 10 depending on the sensitivity required for the device [113]. Rationally, the lower the blank signal noise and bandwidth (blank signal noise average and standard deviation) due to effective optical integration, the lower the potential device *LOD* that can be attained. As such, to achieve optical detection performance comparable to established laboratory approaches, progress is required to provide efficient optical pairing with these microfluidic systems.

In this section we aim to assess the recent approaches in OMLoC integration to overcome the physical constraints, achieving both sensitive and stable detection methods with resultant high *LOD* performance. From here, we aim to identify the progress and remaining challenges to apply these devices into real-world OMLoC applications to form low-cost, sensitive and continual throughput analytical devices.

### 3.1. Absorbance

UV-Visible (190 to 750 nm) absorbance detection is a common, label-free analytical technique where wavelength dependant transmission is determined to calculate absorbance. Photonic interaction can therefore be used to identify and quantify analytic compounds. The low sample requirements, non-invasive nature and potential for continuous flow analysis with mono and multi-phase microfluidics have made this approach of constant interest for lab-on-chip devices. While initially integrated as a simple detection method for μ-scale electrophoresis and chromatography devices [114], this approach has become a fundamental detection method in a multitude of μ-scale sensing systems.

On the one hand, the conversion of transmission into absorbance is governed by a logarithmic transformation as seen in Equation (18). On the other, the Beer-Lambert law regarding the absorbance demonstrates the correlation between species molar absorptivity (ϵ), molar concentration (*c*) and optical path length (*l*), all being essential factors when determining sample optical attenuation.

(18) $$A=ln(\frac{I_{0}}{I})=\epsilon cl$$

Absorbance (*A*) through logarithmic conversion of transmittance, *I*_0_ being light intensity without sample, *I* being detected transmittance through a given sample.

**Figure 16:**
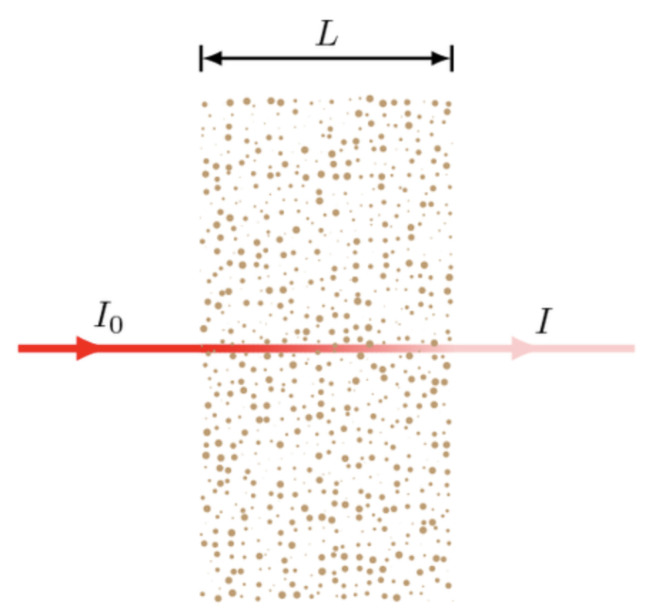
Representation of absorbance detection, where $I_{0}$ represents the initial light input, *I* represents the resulting transmitted signal and *L* being the optical path length.

The relation between the absorbance, transmittance and the Beer-Lambert law equation factors are illustrated in Figure 16, highlighting the requirements for effective absorption detection.

Sample analysis with low molar concentration (*c*) meaning low absorption (*A*) requires a high optical path length (*l*) in order to provide sensitive absorption detection. Optical path length therefore remains a constraint to sensitive analysis, which at first glance, is not compatible with μ-scale devices. In addition, stray light presence can similarly reduce the detection performance [115] with ambient light leakage causing deviations from the Beer-Lambert law. As such, recent efforts have focused on resolving both optical path length and effective photonic pairing to increase device sensitivity and stability. However, beyond (i) optical path length, there are several other criteria to facilitate UV-Visible spectroscopy within micro-scale systems, such as (ii) material UV-Visible light transmittance and (iii) optical guiding to further reduce optical attenuation. This section aims to address the innovative ways in which UV-Visible detection is being integrated to overcome these apparent constraints.

#### 3.1.1. Optical Path Length

**Figure 17:**
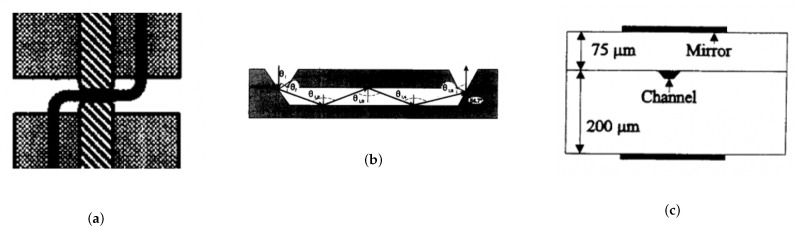
Initial approaches to overcome path length of on-chip absorbance detection (**a**) Z-shaped flow cells. Reprinted from [114]. Copyright © Elsevier 1991. (**b**) Silica multi-reflection cell. Reprinted from [117], copyright © Elsevier 1992. (**c**) Multi-reflection cell in glass with aluminium plating. Reprinted from [118], copyright © Electropheresis 2000.

To address the challenges of optical path length, early μ-scale systems used Z-shaped channels [114] with longitudinal transmission through silica capillaries (Figure 17a). In this way, increased photonic sample interaction provided a six fold improvement in the signal to noise ratio with a 3 mm path length meaning higher sensitivity in applications such as capillary electropheresis. Moreover, the integration of silicon micro-fabrication and longitudinal detection also saw U-shaped channels in planar glass chips with integrated optical fibers [116], increasing longitudinal transmission pathways up to 140 μm. Again proving, counter-intuitively, that optical path length could be augmented at the micro-scale.

Another method to augment the optical path length while maintaining the same physical channel dimensions was achieved with the use of multi-reflection cells [117]. Silicon micro-fabrication was used to develop an in-plane optofluidic waveguide (Figure 17b) for use in absorbance spectroscopy. Here, optical angle input control can increase the number of reflections and therefore the theoretical path length to increase the device sensitivity. Multi-reflection cells were then also developed using glass plates with aluminium for simpler fabrication (Figure 17c) [118] and increased material reflectance for optical confinement, demonstrating 5 to 10 fold path length enhancement (50–272 μm) compared to single pass devices (10–30 μm).

**Figure 18:**
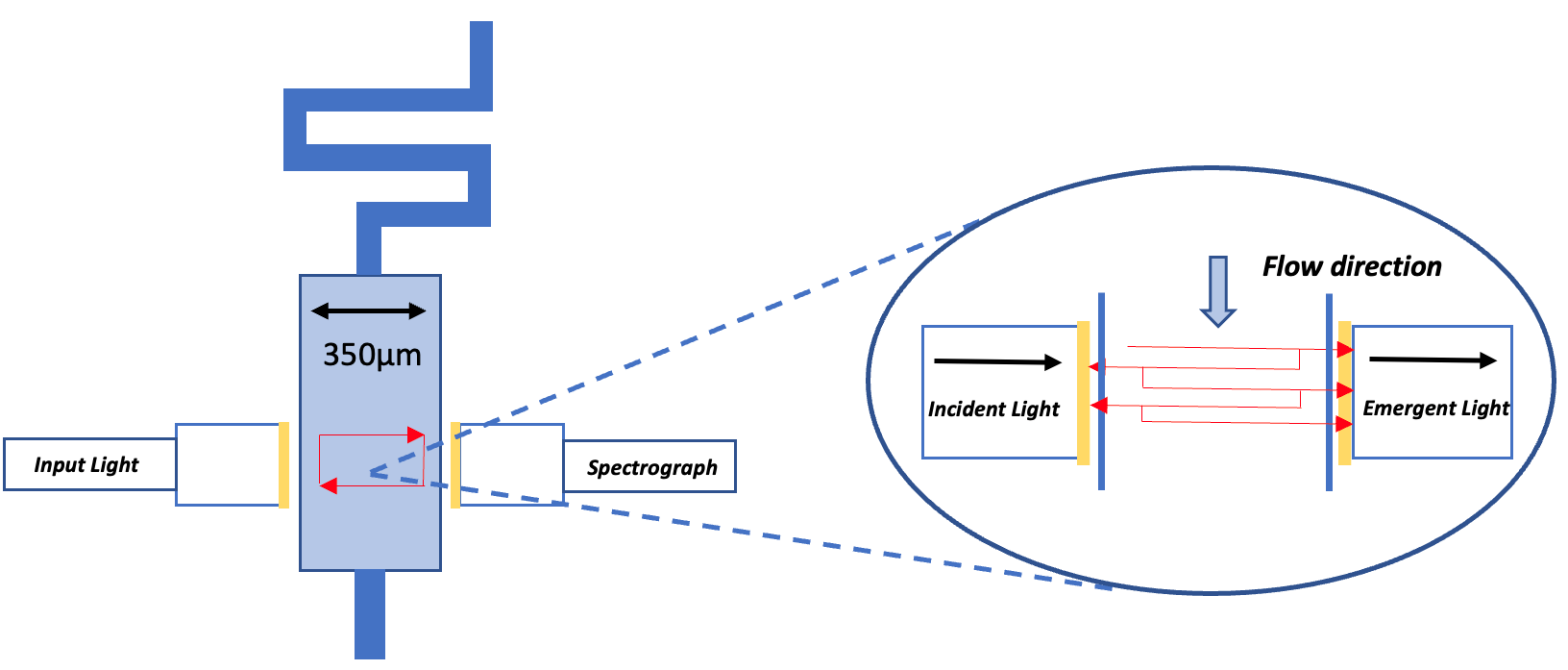
The use of gold-coated optical fibers as a F-P resonator for microfluidic detection. Integrated device with microfluidic reactor prior to the detection channel, plus schema of the F-P resonator traversing the detection cavity, augmenting the theoretical optical path length. Illustration adapted from Zhu et al. [119]. Printed with permission from Zhu et al. (2017). © 2017 Royal Society of Chemistry.

Again using reflection to increase the theoretical optical path length, Fabry-Perot (F-P) resonators formed through 2 parallel gold-coated optical fibers that traverse the detection cavity [119] have been recently demonstrated to achieve real-time phosphate detection (Figure 18).

In this transversal approach, effective optical path lengths up to 900 μm could be achieved, compared to the physical resonator length of 300 μm due to multiple light reflections within the F-P resonator, achieving a LOD of 0.1 μmol/L. This demonstrates an adapted optical technique that can be integrated within soft-polymer microfluidics to extend optofluidic interaction length. However, fabrication and alignment of the gold-coated fibers remains technically challenging, limiting wider usage.

#### 3.1.2. Material Transmittance

Another criteria for UV-Visible spectroscopy integration is the transmittance of the material used to fabricate the OMLoC device. The material must have little to no optical attenuation through scattering or absorbance to ensure highly sensitive detection. However, materials with high levels of UV transmittance tend to be both expensive and challenging to fabricate, as seen in Table 1.

In recent years there has been an increase in polymers and thermoplastics to democratise microfluidics through cost-reduction and simpler fabrication processes [6]. However, these materials have generally reduced optical quality compared to the more expensive counter parts. The OMLoC material choice therefore remains essential when developing a novel detection system, balancing the optical quality with the overall cost of device development and the eventual possibility of large-scale production.

**Figure 19:**
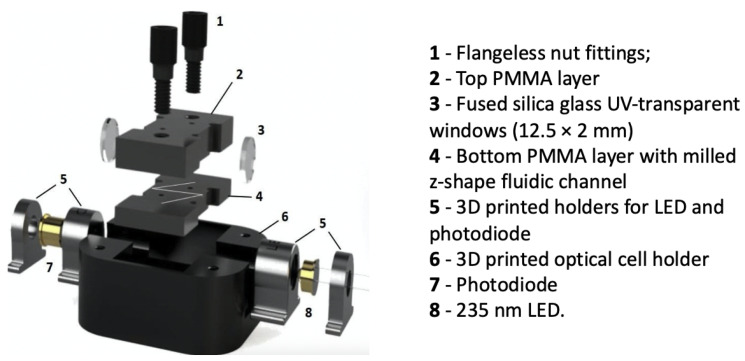
Integrated fused silica windows with a PMMA microfluidic device for UV-Visible absorbance detection. Reprinted from [123], copyright © Elsevier 2019.

Early examples of μ-scale devices formed through silicon etching used thin layers of ${\mathrm{Si}}_{3}N_{4}$ as optical windows [117] to pair light into the micro channel. However, the manual bonding of separate layers of the silicon device was both process/resource intensive and challenging regarding optical alignment, thus reducing optical efficiency. Recent examples of integrating optical windows within lower-cost microfluidic systems are seen with the use of fused silica glass windows (12.5 mm × 2 mm) in contact with the low-cost PMMA microfluidic device [123], as seen in Figure 19. The effective device path length was calculated at 96% of the actual optical path (2.15 cm) meaning low-level scattering, potentially due to the non-transmittance of the deep-UV (235 nm) within the PMMA.

As mentioned, the transition into polymer and thermoplastic based microfluidics means a reduction in cost while increasing LoC prototyping capabilities [6]. This integration of polymers has meant novel fabrication challenges for photonic integration. For instance, PDMS, the current most common polymer for microfluidics prevents UV transmission below 380 nm [124]. This lack of UV transmittance was challenged when Ma et al. [125] used a 100 μm PDMS window to achieve high transmission levels down to 210 nm. The use of thin layers of PDMS separating the detection cavity have then been used in multiple examples of optical fiber integration [119,126]. Additionally, a hybrid approach using quartz, PDMS and SU-8, an epoxy-based negative photoresist, to form a whole channel imaging detection (WCID) [127] for capillary electropheresis was used to form an integrated optical slit to increase sensitivity. Low-cost optical slit formation was achieved with SU-8 meaning reduced alignment issues, showing a bridge between high-cost optical materials and polymer-based microfluidics.

#### 3.1.3. Optical Waveguiding

To improve optical integration and OMLoC performance, photonic waveguiding has been adopted within multiple approaches to ensure light confinement and targeted delivery to the micro-sample. In this way, the refractive index difference between the core and that of the cladding can be used to achieve optical waveguiding, in a similar manner to that of optical fibers. Multiple formats have been explored such as (1) solid-core, solid-cladding [128], (2) liquid-core, liquid-cladding [129] and (3) liquid-core, solid-cladding [130], waveguides.

To simplify polymer-based waveguide formation, complete-PDMS waveguides were first demonstrated with RI alteration through PDMS cure time [128]. An increased cure temperature of 150 °C for 60 min increased the RI to 1.47 compared to 1.45 attained at room temperature, achieving low light loss propagation of 0.4 dB/cm at 460 nm. In addition, varying PDMS to curing agent ratios [131] have been shown to control the refractive indices, again facilitating waveguiding. Furthermore, the use of air mirrors to facilitate internal reflection within PDMS waveguides [130] have been used to achieve strong levels of absorbance detection down to 41 nM with a diluted fluorescein solution. As such, novel physical RI property alteration in polymer-based materials enables a simplified optical integration approach for OMLoC development.

**Figure 20:**
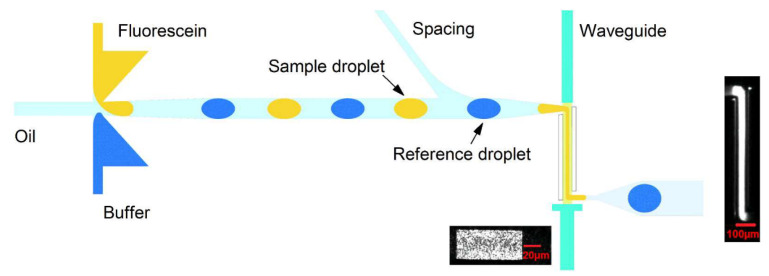
Use of liquid-core optical waveguiding through low-cost polymer microfluidics in PDMS, facilitating droplet analysis in the KHz range. Reprinted with permission from [126]. Copyright (2017) American Chemical Society.

Also, an adapted method to achieve optical waveguiding can be seen through using PDMS liquid-core waveguides with high RI immersion oil (1.515) aligned with the detection channel [126], as seen in Figure 20. In this case, reduced analytical channel diameter also increases the interaction between the whole sample and the coupled light, thus enhancing device sensitivity with an LOD of 400 nM for aqueous solution of fluorescein. Plus, uncured PDMS was set on the device sides to reduce scattering with optical pairing into the PDMS, demonstrating a novel method to reduce optical attenuation with polymer microfluidic photonic coupling.

As previously mentioned, the combination of optical detection with multi-phase droplet flows [7] for reaction monitoring is of rising importance. These efforts build on the pioneering work of Nguyen et al. [132] where multi-phase flows could be identified through integrated optical fibers within a pre-fabricated PMMA-based device. As seen in Figure 20, the current ability to detect absorbance measurements at the KHz range with a single microfluidic device is an essential step towards both highly-sensitive and high-throughput analytical performance promised by the LoC paradigm.

Monolithic waveguides again present an alternative formation approach, providing simpler and increasingly low-cost waveguides that also function through index-guiding. For example, femtosecond laser ablation of glass/fused silica [11] results in waveguide formation with altered local refractive indices, meaning mask-less local waveguide formation. This method has since been applied into other polymeric substrates, such as PMMA, making the approach more integrated with established microfluidic techniques [133]. Channel formation has been achieved in aerogel, a highly porous material (98% air) with a refractive index of around 1.06, enabling TIR within a fluid sample [134]. The development of a micro-photoreactor within an aerogel [13] highlights an effective optofluidic confinement. While silica aerogels are renowned for their fragility, the use of more durable hybrid aerogel-like xerogels that can be dried at ambient pressure [135] could render these materials more suitable for detection applications.

**Figure 21:**
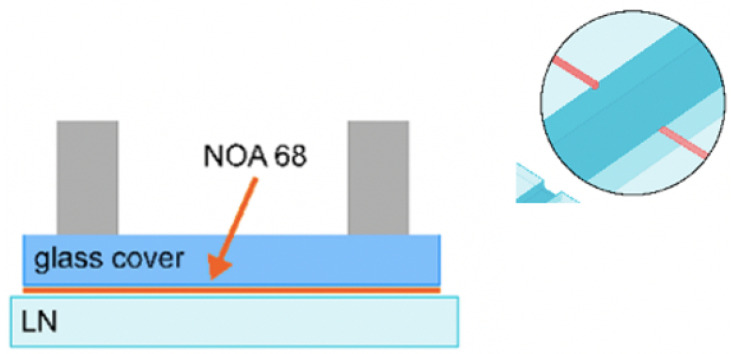
Low-cost Li:Ti waveguides for transmission detection. Reprinted from ref. [136].

Lithium niobate (${\mathrm{LiNbO}}_{3}$) is also of increasing interest for the formation of self-aligned, monolithic optical waveguides and microfluidic channels [12]. Waveguides are implemented in ${\mathrm{LiNbO}}_{3}$ through localised titanium (Ti) doping that is used to change the local RI. A standard photo-lithographic process is used to control the dimensions of the Ti doped waveguides, which permits the fabrication of multiple guides and geometries at a time. These efforts have been recently shown to measure transmittance through a fluidic sample [136] (Figure 21) for pH detection and therefore hold potential for UV-Visible spectroscopy. The current set-up relies on butt-coupled optics through objective lenses that suffers inevitably from background transmission noise, however the team have proposed pig-tailed optical fibers that would provide a simple monolithic, integrated optofluidic solution.

In terms of low-cost microfluidic based detection, a recent important development is seen with industrial production of deep-UV light-emitting diodes LEDs (235 nm), as used by Murray et al. [123]. A stable, low-cost UV light source at a fixed wavelength could in many ways act as a catalyst for the development of similar sensitive and stable detection devices. UV LED integration alongside high-quality optical material such as fused silica windows and waveguiding to increase the optical integration efficiency present enormous potential for numerous industries and applications requiring sensitive detection.

### 3.2. Luminescence (Fluorescence/Chemiluminescence)

**Figure 22:**
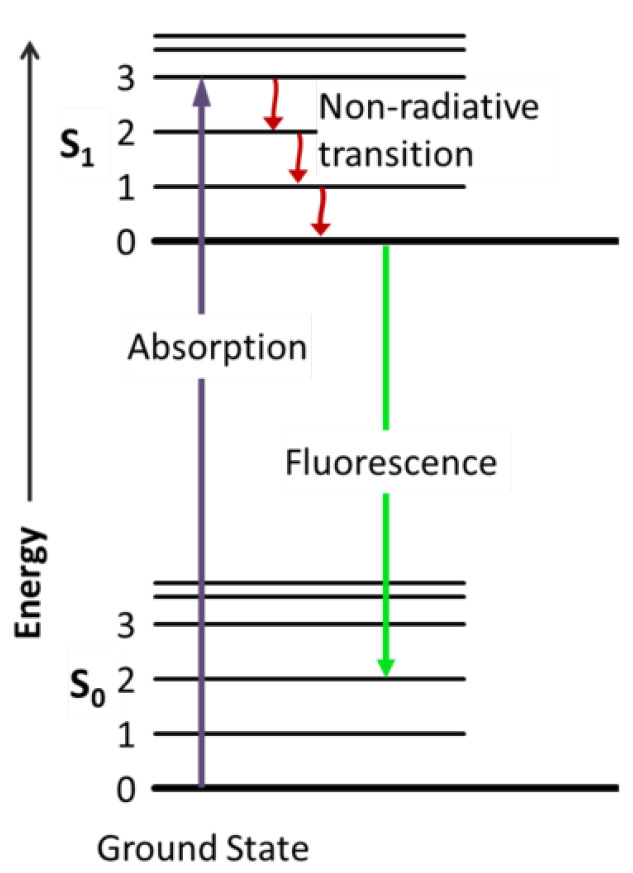
Jablonski diagram of changing electron states that enables fluorescent detection. From initial photonic absorption, through transition and eventual lower-energy photonic transmission.

Fluorescence detection relies on a 3-stage process of excitation, excited-state lifetime and transmission of a fluorescent dye, being either a molecule (auto-fluorescence) or a material (fluorophore, quantum dot), binding to proteins, nucleic acids or lipids within a sample. Photonic absorption takes place resulting in an excited electronic state. The excited state lifetime then causes a delay and energy loss before the fluorophore transmits photonic energy at a lower overall frequency that can be easily determined, as seen in Figure 22.

The high signal to noise ratio provides the highest sensitivity and selectivity for optical detection [112], enabling single molecule detection. In addition, varying fluorophores can be used to achieve multiplexed detection. Resultantly, there has been a continued demand for OMLoC fluorescence integration with mono and multi-phase microfluidics for analytical purposes.

**Figure 23:**
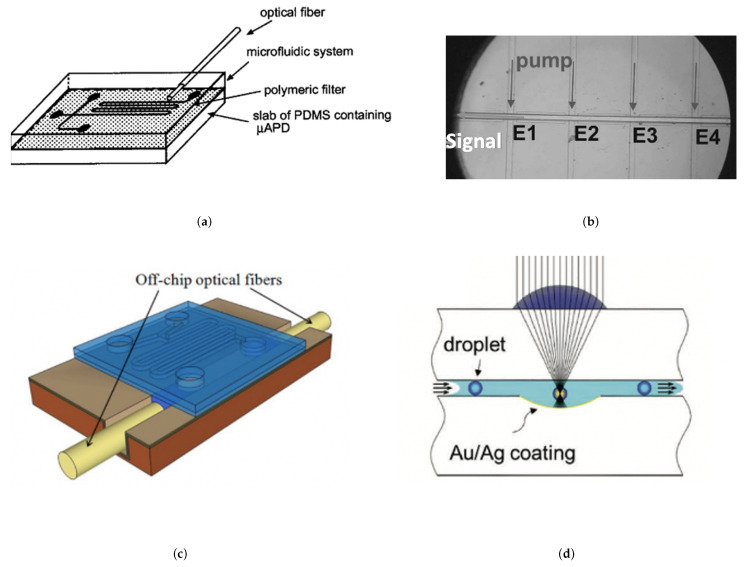
Approaches to achieve OMLoC fluorescence detection. (**a**) integrated fluorescence detection in PDMS. Reprinted with permission from [138]. Copyright © (2001) American Chemical Society. (**b**) Hollow and solid ARROW waveguides. Reprinted with permission from [139] © (2006) The Optical Society. (**c**) PDMS and ARROW integration. Reprinted with permission from [9] © (2014) The Optical Society. (**d**) Integrated co-axial mirror and lens system within a PDMS device. Reprinted from [140], copyright © (2020) John Wiley and Sons.

However as sample volume increases, the background noise due to sample impurities also increases meaning a reduction in the fluorescence signal per molecule/background noise. Therefore with fluorescence (contrary to absorbance), the reduction in detection volume reduces noise originating from both Rayleigh straylight and raman scattering [137] that can lower the device sensitivity and therefore the LOD. As such, confined devices with short optical pathways for analysis are preferred due to augmented sensitivity. Thus, the challenge is to form these small scale systems that are also capable of high-throughput microfluidic capabilities for LoC applications. Figure 23 demonstrates several recent approaches to achieve OMLoC fluorescence detection.

A simple, initial approach saw an integrated multi-mode fiber to provide optical illumination of multiple PDMS channels [138] with an embedded μ-avalanche photodiode (PD) to detect emission, seen in Figure 23a. An 80 μm poly-carbonate filter was used to absorb scattered excitation light before reaching the detector. Here, the sample being interrogated is around 0.15 nL due to focused light across a partial section of the micro-channel. While fluorescein detection was demonstrated at 25 nM, collection efficiency remained low at 0.2%. From this early stage it was suggested that optical wave-guiding could improve collection efficiency and improve the LOD.

Anti-resonant reflective optical waveguides (ARROW) with alternating dielectric layers represent another popular system for optical confinement and have remained promising for fluorescent detection [141]. The micron sized channels are ideal for realising on-chip fluorescence single particle detection, as achieved by Yin et al. [139] with sub-pL sample volumes, where a direct photo-multiplier tube attached to the device simplifies the method of detection. Here, the use of solid and liquid core ARROW waveguides facilitated a planar approach to optofluidic guiding to achieve highly sensitive fluorescent detection down to the particle scale, Figure 23b. However, complex fabrication requiring plasma-enhanced vapour deposition has limited their application to mainly proof-of-concept devices. Also, while dramatically reduced, light loss from ARROW devices is still a factor due to the leaky mode nature of the interference methods used to contain the light.

Recently, PDMS/ARROW composite devices have been demonstrated to form disposable fluidic units with PDMS [9] to integrate soft-polymer microfluidics with the more complex ARROW devices. As seen in Figure 23c, these devices provide sufficient optical confinement and integrate with established microfluidic methods, enabling an LOD down to 2.5 nM of Cy5 dye. This approach has led to breakthroughs of single nucleic acid fluorescence detection of Ebola within clinical samples, demonstrating the potential applications in amplification-free point-of-care (POC) detection [142]. Specifically for POC diagnostics, the capability to dispose of the fluid handling part of the device could be advantageous due to issues with cross-contamination. While in some ways a bridge with polymer based microfluidics, these systems still require complex fabrication to achieve alignment and therefore currently remain far from an industrialisable POC diagnostic solution.

Another approach has used hydrodynamic focusing within a complete PDMS device [143], to focus the particles within the channel centre to homogenise particle speed and reduce the coefficient of variation (CV) of signal intensity (calculated by dividing the standard deviation of the signal intensities by the mean of the distribution). By delivering the analytes to the area of high light interaction, this improves the efficiency of the device for optical analysis. Here, solid PDMS optical waveguides with an increased refractive index to guide the light into the microfluidic channels have been utilised. The reduction in CV of signal intensity allowed for the first time the detection of individual virus particles with the use of fluorophores in a soft polymer microfluidic device.

In addition, monolithic parabolic mirrors and printed lenses have recently been fabricated above/below microfluidic channels respectively [140] through 2-photon polymerisation. This both improves sensitivity and high-throughput multi-phase droplet detection through enhanced photon excitation and collection, as seen in Figure 23d. The use of both components has enhanced fluorescent signal by 2 orders of magnitude, enabling pL droplet detection at rates of 40,000 per s. Furthermore, the usage of camera phone detection represents a low-cost, high precision method for droplet fluorescence as recently demonstrated [144] with 1 million droplets/s being detected simultaneously through 120 channels. Here, a micro-droplet mega-scale detector and LED source are combined within a simple platform that does not require expensive analytic equipment. Overall, high sensitivity levels combined with novel imaging methods such as camera phones demonstrates an optical method that has great potential with integrated optical detection.

However, fluorescence still requires sample processing for fluorescent tagging and generally the use of wavelength filters for detection. Another approach is chemiluminescence, which represents a simpler luminescence detection method as molecule excitation takes place following a chemical reaction, therefore without requiring photonic input for excitation [112]. Chemiluminesence has been demonstrated in miniaturised systems as a method of detection, for example with fluorescent quenching to determine oxygen levels [145]. Highly sensitive detection is required however which again places a limitation to integration.

### 3.3. Refractive Index Variation Detection

The refractive index is a basic physical property determined by comparing the light velocity through a medium to the speed of light within a vacuum. In the same way, light can be used to determine analytical changes in fluid samples, as seen in Equation (19). Analyte presence within a sample (*s*) induces detectable and scaling RI changes seen with the alteration of light speed compared to the sample blank (*b*), meaning a simple and low-cost method to determine biochemical differences.

(19) n=b/s

with *n* being the refractive index and *b* & *s* being the light phase velocity through a blank and sample respectively.

With high sensitivity, minimal preparation and non-destructive nature, it is clear how RI has remained a central OMLoC analytical method. Approaches to date have included Photonic Crystal Fibres (PCFs), planar ARROW waveguides and WGMs [146]. The approach is particularly attractive for small detection volumes (μL to nL samples) as RI related signals increase along with both bulk concentration and surface density [8]. The integration of μ-channels to bring both static and mono-phase fluidic sample into contact with these waveguides presents a promising, potentially simpler method of fluidic integration, particularly through soft-polymer based microfluidics.

**Figure 24:**
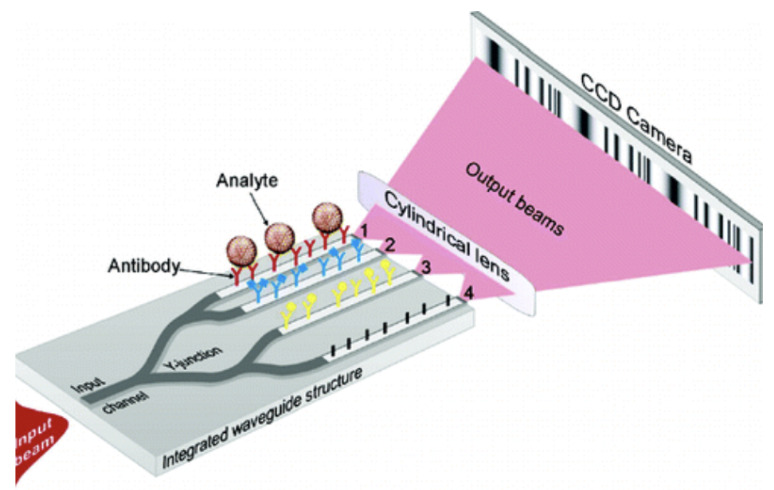
Integrated Young Interferometer with microfluidic approach. Laser light is coupled into the double sample and reference waveguides and phase change due to adsorption is detected through signal integration. Reprinted with permission from [147]. Copyright © (2007) American Chemical Society.

Optofluidic interferometry has remained a common technique for RI change detection, where light is coupled into sample and reference waveguides. Analyte waveguide adsorption results in a phase change of the evanescent wave, detectable as the light is coupled out from the waveguides. An example of which can be seen in Figure 24.

In terms of signal detection, Young interferometers generate an interference pattern as highlighted in Figure 24. These devices therefore ideally require long-interaction pathways to encourage an increase of analyte adsorption across the samples. An early demonstration saw 4 integrated waveguides function as a Young Intereferometer for use as an immunosensor [147]. Virus particle detection of herpes with a minimal concentration of 850 pp/mL [147] were identified, meaning clinically relevant concentration detection. This device provided the possibility of multiplexed detection due to being able to graft 3 different receptors on each of the 3 sample waveguides to be compared with the reference signal. The fast response time of detection (within minutes) and a highly sensitive LOD of around a single virion being possible. Resultantly there has been a focus on interferometry developments for bio-sensing [111]. However, the waveguide receptor grafting process means additional complexity regarding device formation.

Mach-Zender interferometers (MZI) instead recombine the beams and photonic intensity is measured generally through a photo-detector, as seen in Equation (20).

(20) $$I=I_{1}+I_{2}+2\sqrt{I_{1}I_{2}}cos\left(\Delta \phi \right)$$

with *I* being the signal intensity, $I_{1}$ the light velocity of the reference transmission, $I_{2}$ light velocity of sample transmission and cos(Δϕ) being the phase shift difference. Demonstrating that *I* is not only dependant of the intensities of the constituting waves but also of the phase shift that exists between the different waves.

**Figure 25:**
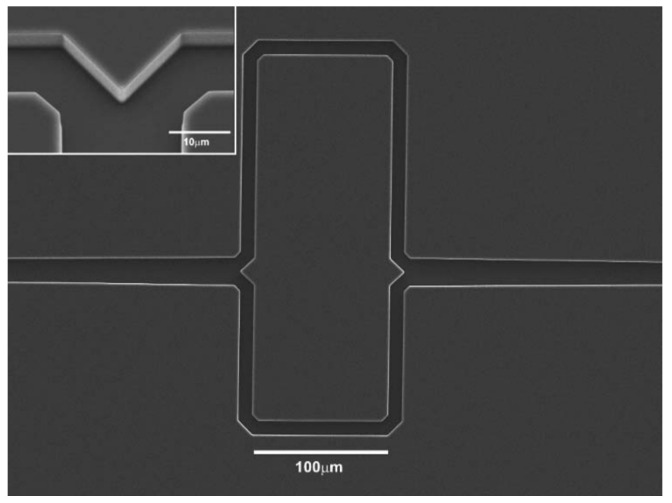
Recent approaches to achieve OMLoC RI detection with an ARROW-based MZI. Reprinted with permission from [148] © (2010) The Optical Society.

RI change detection has also been achieved using established planar optofluidic wave-guiding such as with ARROW waveguides used to form MZI [148], (Figure 25). Here, the light and the sample share the same path, increasing device sensitivity compared to evanescent based devices which are limited by the evanescent fields range per unit length. The device footprint is greatly reduced, requiring just 0.16 nL of sample over a 1 mm length waveguide. Also, the addition of multiple cladding layers can reduce the propagation losses further. However as previously mentioned, ARROW waveguides require chemical vapour and atomic-layer deposition of silicon dioxide and titanium dioxide respectively for the alternating F-P layers, meaning again a complex and high-cost fabrication process.

A simplified fabrication process for MZI was then demonstrated through integrated waveguide and microfluidic channel production in fused-silica using femtosecond laser processing [149]. Reduced irradiation intensity results in smooth surface refractive index transitions for the optical waveguides that can be partnered directly to the microfluidic channels. Optical fibers can then be integrated at either side of the waveguides, providing a comparatively simpler and attainable LoC detection system. Pairing the device with a capillary electropheresis chip, the team demonstrated peptide detection of ±10 mM. However, laser processing can result in sub-optimal microfluidic channels due to rough inner surfaces that could limit potential future applications. An alternative approach is seen using self-trapped light beams to induce low loss buried waveguides in (${\mathrm{LiNbO}}_{3}$) [150]. In this case, visible light is paired into the substrate to induce circular waveguides through altering the refractive index through a photorefractive effect. A transverse milled microfluidic channel can then be used for sample refractive index detection. This approach provides promise for reversible waveguide integration, that could potentially be used with photosensitive polymers.

**Figure 26:**
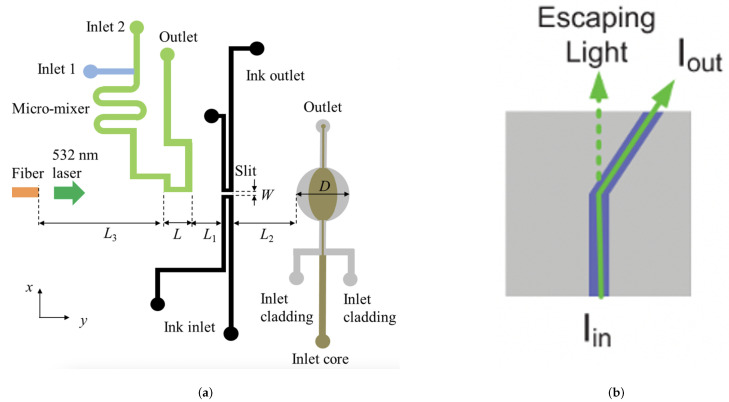
(**a**) PDMS based optical slit system. Reprinted with permission from [151] © (2019) The Optical Society. (**b**) Bent waveguide structures using SU-8. Reprinted with permission from [152] © (2018) The Optical Society.

Also, microfluidic ink channels have been used as optical slits in PDMS, with an optical fiber and integrated optofluidic lens [151], presenting a low-cost, sensitive detection approach, seen in Figure 26a. Here, the use of diffraction imaging means an RI change detection of around 10${}^{-5}$ RIU through detecting optical phase change induced by analyte presence. The 330 μm optical slit and dual sample input with passive mixer, allows continuous sample input meaning faster and higher throughput analysis. The authors predict that with the use of a 2D image sensor array, multiple chips can be read to enable high-throughput for on-site detection.

Finally, a recent method to simplify device fabrication has been the use of bent waveguide structures [152] on low-cost glass substrates using SU-8, seen in Figure 26b. The detection of output light intensity variations from the straight to bent waveguide is highly representative of RI differences of contained solutions with impressive RI resolution over a wide dynamic range of 0.04 RIU. The use of cyclic olefin co-polymer, integrated LED and photo-detector demonstrates a low-cost and comparatively simple to fabricate RI detection system that is capable of real-time continuous sample monitoring. In addition, the low standard deviation of the output light intensity (0.21%) suggests high reliability of the bent waveguide approach.

### 3.4. Summary

Clearly, OMLoC devices have begun to fulfil the original μ-TAS concept of enhanced sensitivity [142] with reduced reagent/sample fluid consumption [8]. Photonic integration has meant surpassing the boundaries of traditional photonic detection to the particle level [153]. However, these approaches remain mostly proof-of-concept, closer representing “chips in a lab” than “lab-on-a-chip” [154]. Both high cost and fabrication complexity often detracts from their original goals of a device non-reliant on expensive lab equipment.

While micro-fabrication has transitioned to polymer-based devices through soft-lithography, fabrication requirements still need specialist equipment and training, for example integrated ARROW/PDMS devices [155]. Therefore, the development of monolithic waveguides [11] are an interesting alternative, overcoming complex micro-fabrication techniques. Additionally, analytic modules that can be incorporated in pre-existing workflows could represent a bridging step towards micro-scale optical detection. These novel approaches could overcome short-term fabrication challenges, specifically by using novel additive manufacturing processes such as 3D printing.

The demand for low-cost, sensitive detectors is increasing exponentially with Internet of Things (IoT), cloud-based computing and the increased demand for continuous monitoring in applications such as healthcare and water monitoring. Furthermore, high environmental and financial risks of pollution [123] and biological crises [156] require monitoring and mean research budgets must increase to counteract these growing risks. Resultantly, we can expect that OMLoC devices will continue to shift from demonstrating potential to demonstrating their applicability in these increasingly challenging domains.

## 4. Perspectives and Trends

### 4.1. Materials

**Figure 27:**
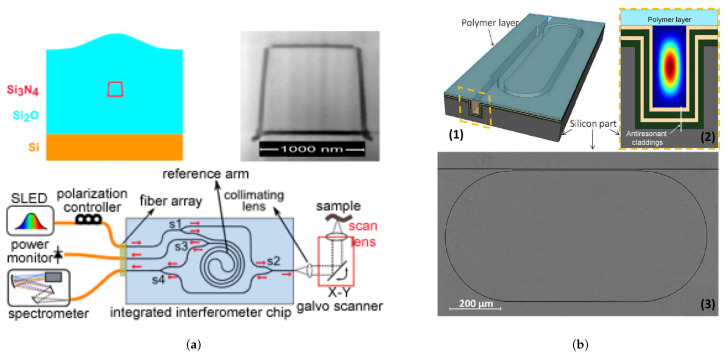
(**a**) Interferometric MZI using an ${\mathrm{Si}}_{3}N_{4}$ and ${\mathrm{SiO}}_{2}$ waveguide technology called $TriPleX^{TM}$. Reprinted with permission from [157] © The Optical Society. (**b**) ARROW based Ring resonator sensor: (**1**) a 3D schematic view of the liquid-core ring resonator, (**2**) a cross section of the hybrid ARROW, showing a simulated power distribution of the fundamental mode, and (**3**) an SEM photo of the silicon bottom layer supporting the antiresonant cladding. Adapted from [158], with the permission of SPIE.

We can see a clear trend towards using silicon processing technologies for photonics with PDMS for microfluidics [76,78,84,88]. Silicon processing technologies offer good quality waveguides, high refractive index contrast and a large spectrum of transparency to electromagnetic waves, depending on the dopants. The high RI contrast offers the possibility to implement extremely complex designs with small bending radii hence with a reduced on chip footprint [157] as seen in Figure 27a.

ARROWs are also compatible with standard silicon processes since they employ typical dielectric materials such as SiO${}_{2}$ and Si${}_{3}$N${}_{4}$ for cladding deposition in order to create the hollow core waveguide [9,141,159,160] as depicted in Figure 27b. Their propagation losses are not affected by bending [161] and hence can be employed for complex circuitry as it is the case for high RI contrast waveguides. They also have the ability to channel light and fluids in their hollow core which offers a more important interaction between the two entities and this is especially interesting for sensing applications. At the same time, PDMS along with other polymers such as PMMA and SU-8, remain the materials of choice for the microfluidic channels fabrication. Yet the alignment process is quite challenging when working with PDMS and PMMA, which is not the case for photosensitive polymers such as SU-8 for example.

**Figure 28:**
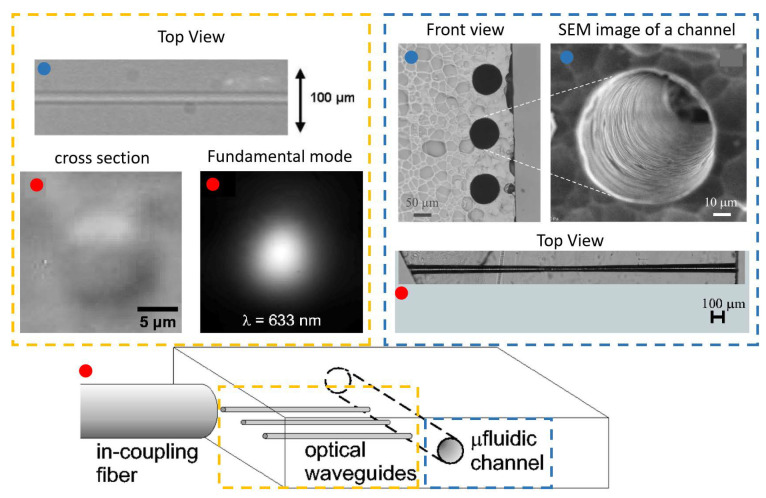
Femtosecond fabricated optofluidic platform for parallel multiple sensing at different positions in the micro channel. Details of channels and waveguides that can be fabricated in glass using femtosecond laser processing are illustrated separately. These images are adapted from multiple works of Bellini and Osellame using similar device formation. Marked with a red dot [164]. Marked with a blue dot [165]. Adapted from [164,165], with the permission of AIP Publishing.

Another trend is the use of femtosecond laser processing in order to both induce a refractive index increase in glass [86,108] and polymers [162,163] on one hand, and microchannels in glass through Femtosecond Laser Irradiation followed by Chemical Etching (FLICE) and in polymers through laser ablation [133] on the other hand. The ultimate power of this relatively new technology resides not only in its ability to produce monolithic OMLoCs but in its capacity to produce 3D microchannels and waveguides as illustrated in Figure 28. Its only weakness is its high energy consumption in order to tightly focus the intensity of the beam to produce multiphoton absorptions in a specific geometrical point. As a matter of fact, nonlinear absorption in glasses takes place for intensities around 10${}^{13}$ W/cm${}^{2}$, for a pulse duration of 100 fs.

**Figure 29:**
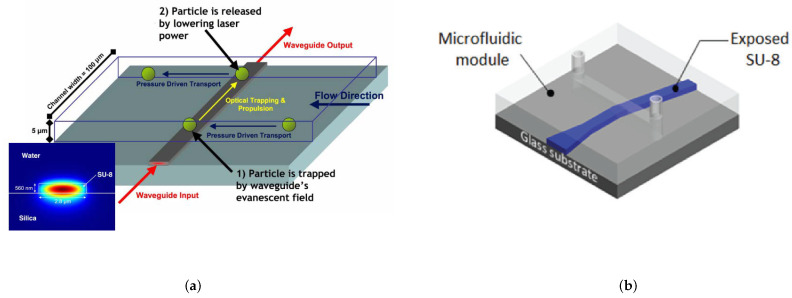
(**a**) SU-8 fabricated waveguides for trapping platform. Adapted with permission from [78]. © (2007) The Optical Society. (**b**) SU-8 fabricated waveguides for sensing platform. Reprinted with permission from [152]. © (2018) The Optical Society.

On the other hand, “all-polymer” based optofluidic platforms have been demonstrated to be attractive due to the ease of processing and low fabrication cost. These approaches are especially interesting if the polymers are photosensitive, such as the case for SU-8 in which the fabrication of low cost waveguides by refractive index change has been demonstrated [78,152,166] as we can see in Figure 29. In addition, the simple fabrication of all-PDMS waveguides through introducing PDMS with varying curing ratios [131] and the use of PDMS/air waveguides to achieve TIR [130], again represent potential in this field. However, polymer waveguides are known to suffer from high propagation losses.

In order to democratize this field, there is a need to extend the catalogue of materials used for OMLoC implementation. This necessity arises from the market’s high demand, which should be addressed with complementary materials that are compatible with the silicon processing technologies and which offer a wider choice of specifications than those currently available. There is an interest in materials that offer the high quality of silicon and glass platforms and the ease of polymer fabrication. For this reason, hybrid organic-inorganic photoresists [167,168] and ultra porous materials [13] have a great potential in this field.

### 4.2. Modular Optofluidics

An increasingly popular fabrication method is through standardised modules for LoC applications. These approaches facilitate precise analytical devices with reduced fabrication costs while being functional for untrained users. An example being the hybrid integration of separate fluidic PDMS and photonic ARROW layers to perform fluidic management and detection simultaneously [9]. These discrete modules could potentially overcome the lack of a simple and universally established OMLoC fabrication process.

A modular optical unit has been recently demonstrated with a micro-milled LED system that encapsulates Polytetrafluoroethylene (PTFE) tubing in order to perform high-throughput absorbance measurements on droplets [169]. In this case, four detection cells are formed through LEDs in micro-milled holes. The light path distance of 700 μm of the PTFE tubing is sufficient for absorbance measurements and the multiple detection points mean the system can detect droplet size and velocity with an inter-detector absorbance sensitivity variation of 3.3%. Colorimetry is then used as a proof-of-concept to demonstrate a glucose enzymatic reaction, showing that absorbance is linear for all concentrations at a given time. The flow cell and PTFE tubing usage overcomes current issues associated with complex micro-fabrication while also presenting a low-cost approach to droplet analysis.

Microfluidic modularity has been furthered by PDMS ’Lego-like’ modular blocks [170] to form 3D microfluidic networks. From this, micro-milled LEGO® bricks for a “lab-on-brick” modular approach [171] have been developed. For optical detection, an infrared light source (940 nm) and a PD sensor attached to L-shaped bricks were paired on the opposing sides of transparent fluidic bricks, where the LEGO mounts ensure alignment. Analysis of acute signal differences meant droplets could be identified. Increased transparency and light wave-guiding would then further optical detection sensitivity. The approach promotes open-source, standardised modular detection methods [172]. While still in it’s infancy, this research highlights the move towards democratising novel fabrication methods that could circumvent high cost and complex micro-fabrication.

**Figure 30:**
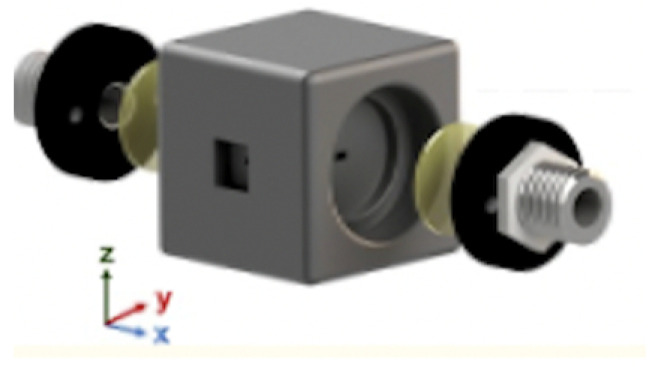
A modular 3D-printed waveguide. Adapted from [174], with the permission of AIP Publishing (2019).

In addition, Microfluidic Instrumentation Components (MFIC) have been recently developed, allowing assembly of discrete modules to form on-demand microfluidic systems. For instance, an open-source library of microfluidic modules that can be easily formed through stereo-lithography [173]. In this approach, an infra-red MFIC detection module was incorporated downstream from a droplet formation module of water in fluorocarbon oil, where a photo-transistor detects photonic absorbance changes for droplet detection and potential analysis. From this, a modular 3D-printed work flow spectrophotometry has been demonstrated [174], with an encapsulated waveguide (36.5 mm in length), seen in Figure 30. The device has been demonstrated with stopped-flow enzymatic detection with equal performance to bench-top assays while also demonstrating low reagent consumption and reduced assay time. These MFIC remain highly promising to integrate within industrial work flows for high-throughput sample detection. The increasing availability of high-precision additive manufacturing techniques such as 3D printing could mean the democratisation of these module-based analytical approaches.

## 5. General Conclusions

Evidently to date, there exists no universal fabrication method for optical integration to achieve analysis and manipulation, the two vital functions of the Lab-on-chip paradigm. Modular approaches could represent a promising approach to short-term commercialisation. Also in an effort to escape from the chip in a lab concept, smartphone enabled imaging and detection can be an efficient initial alternative to highly sophisticated laboratory photonic equipment.

As we have seen for optical manipulation, there is a perpetual need to manipulate smaller particles down to the molecular level, while only deploying μW powers. This was enabled mainly by plasmonic structures which come at the cost of a nanometric precision. Waveguide traps on the other hand, offer a low cost and easy to implement alternative especially when fabricated in polymer based platforms.

In terms of analysis, novel OMLoC solutions to optic and microfluidic integration continue attempting to overcome the underlying physical constraints linked with their associated detection modes. In doing so, these devices can achieve highly stable detection with an associated high LOD. Evanescent waveguiding interferometry [147], leading to ARROW di-electric waveguiding interferometry [148] that remains highly sensitive with a fraction of the sample volume represents this progression towards LoC aspirations. While fabrication remains complex, these systems develop understanding of the detection mode at the μ-scale and are therefore likely laying the foundations for simple integrated OMLoC detection modules.

While there has been undoubted progress, optofluidic lab-on-chip remains far from being widely used for manipulation and analysis outside of a lab setting. To date, the use of these devices remains mainly limited to research, due to their custom designs and non standard, non scalable fabrication processes. The development of high throughput, low cost fabrication processes of LoCs and OMLoCs in particular, is essential for both market growth and their contribution to public health mainly through: rapid screening of pathogens and POC diagnostics, especially in low resources settings, and in periods of biological crisis.

Therefore, the identification of simpler and increasingly standardised fabrication methods is a central focus to realise OM-LoC devices. In order to put such a concept to work, an agreement must be reached between the different stakeholders from researcher, to industries all the way to the users. This agreement must set a panel of materials of choice, standardised dimensions of the used substrates and a general guideline for materials and associated fabrication techniques. This ideal approach is not far from reality as it is already being established for the microfluidic community.

Ultimately, the promise of novel physical advantages at the μ-scale such as increased reaction rates, predictable fluid control and increased detection sensitivity that can be theoretically achieved through optical pairing are too great to be missed. While technological optofluidic integration continues evolving to realise these devices, multiple industries and domains stand poised on the verge of a new dawn of analytical and manipulation capabilities.

## Figures and Tables

**Table 1:** Material and physical characteristics of common materials used in OMLoC fabrication (gathered from varying suppliers such as Edmond Optics [120], Neyco Materials [121], Sigma Aldrich [122]) ($\lambda_{min}$ * represents the minimum wavelength (nm) transmitted by the material).

Material	$\lambda_{min}$ *	Refractive Index	Cost (EUR)	Fabrication Method	
Quartz	190	1.55 to 1.54	80 (75 mm diam)	Micro-milling Chemical etching	
Fused Silica	180	1.55 to 1.40	75 (25 mm diam)	Micro-milling Chemical etching	
Borosilicate glass	350	1.51	0, 10 (25 × 75 mm)	Micro-milling Chemical etching	
SU-8 photoresist	400	1.59	7, 5 (10 mL)	Photolithography	
PDMS	380	1.43	4 (25 × 75 mm)	Soft lithography	
Polymethyl methacrylate (PMMA)	400	1.49	2 (25 × 75 mm)	Hot embossing Injection moulding Laser machining	
cyclic olefin copolymer (COC)	350	1.53	12.3 (10 g)	Hot embossing Injection moulding Laser machining	

## Abbreviations

The following abbreviations are used in this manuscript:

LoC	Lab-on-Chip
OM-LoC	Opto-Microfluidic Lab-on-Chip
UV	Ultraviolet
IR	Infrared
OET	Opto-Electronic Tweezer
COT	Conventional Optical Tweezer
ITO	Indium Tin Oxide
DEP	Dielectrophoresis
MST	Maxwell Stress Tensor
SIBA	Self-Induced Back-Action
HHD	Helmholtz–Hodge decomposition
PDF	Probability Density Function
PS	Polystyrene
PDMS	poly(dimethylsiloxane)
WGT	Waveguide Trap
RCT	Resonant Cavity Trap
POT	Plasmonic Optical Trap
nSWAT	nanophotonic Standing Wave Array Trap
SOI	Silicon-on-insulator
WGM	Whispering Gallery Mode
L2	Liquid–Liquid
RBC	Red Blood Cell
RI	Refractive Index
LOD	Limit of Detection
POC	Point-of-Care
F-P	Fabry–Perot
NA	Numerical Aperture
TIR	Total Internal Reflection
PMMA	Poly(methylmethacrylate)
COC	Cyclic Olefin Copolymer
WCID	Whole Channel Imaging Detection
LED	Light-Emitting Diode
ARROW	Antiresonant Reflecting Optical Waveguides
CV	Coefficient of Variation
PCF	Photonic Crystal Fibres
MZI	Mach–Zender Interferometers
FSL	Femtosecond Laser
FLICE	Femtosecond Laser Irradiation followed by Chemical Etching
PD	Photodiode
IoT	Internet of Things
PTFE	Polytetrafluoroethylene
MFIC	Microfluidic Instrumentation Components
MDPI	Multidisciplinary Digital Publishing Institute
